# The Impact of Donating Human Milk on the Health of the Donor and Their Infant: Evidence from Two Systematic Reviews

**DOI:** 10.1016/j.advnut.2025.100581

**Published:** 2025-12-30

**Authors:** Kendall E Baier, Alaina Berg, Abigail Smith, James Evans, Jaimie Rogner, Mohammed H Murad, Tarah Colaizy, Zulfiqar A Bhutta, Aamer Imdad

**Affiliations:** 1 Stead Family Department of Pediatrics, University of Iowa Carver College of Medicine, Iowa City, IA, United States; 2 Health Sciences Library, State University of New York Upstate Medical University, Syracuse, NY, United States; 3 NewYork-Presbyterian Hospital/Weill Cornell Medical Center, New York, NY, United States; 4 Division of Public Health, Infectious Diseases, and Occupational Medicine, Department of Internal Medicine, Mayo Clinic, Rochester, MN, United States; 5 Division of Neonatology, Stead Family Department of Pediatrics, University of Iowa Carver College of Medicine, Iowa City, IA, United States; 6 Centre for Global Child Health, Hospital for Sick Children, Toronto, ON, Canada; 7 Institute for Global Health and Development, Aga Khan University, Karachi, Pakistan; 8 Department of Nutrition, Joannah and Brian Lawson Centre for Child Nutrition, University of Toronto, Toronto, ON, Canada; 9 Division of Gastroenterology, Hepatology, Pancreatology, and Nutrition, Stead Family Department of Pediatrics, University of Iowa, Iowa City, IA, United States

**Keywords:** human lactation, postpartum period, reproductive physiological phenomena, donor human milk, human milk donor, donor infant

## Abstract

Using human milk has been associated with decreased morbidity and mortality in preterm/low birth weight infants. Donor human milk is recommended when maternal milk is unavailable. The benefits of donor human milk for the recipient are well documented, but the impact of donation on donors and their infants is not clear. We aimed to evaluate the effects of donation human milk on donors and their infants. Literature searches were conducted (April 2024) to identify studies (observational, quasi-experimental, and randomized control trials) assessing the impact of human milk donation on donor health, nutrition, well-being, and lactation and on their infants’ health, growth, and development. Bias was assessed using the Risk of Bias in Non-randomized Studies – of Interventions scale. Meta-analysis was conducted when possible. Certainty of evidence was assessed using the GRADE (Grading of Recommendations, Assessment, Development, and Evaluation) approach. Nine studies examined donor outcomes, and 6 studies examined donor infant outcomes. No differences were found between donors and nondonors regarding the prevalence of overweight [risk ratio (RR): 1.27; 95% confidence interval (CI): 0.81, 2.01], postpartum depression (RR: 0.60; 95% CI: 0.21, 1.72), postpartum anxiety (RR: 0.84; 95% CI: 0.59, 1.18), need to pump for their infant (RR: 1.09; 95% CI: 0.63, 1.89), mastitis (RR: 1.48; 95% CI: 0.71, 3.05), chapped/cracked nipples (RR: 0.61; 95% CI: 0.34, 1.12), and breast engorgement (RR: 1.88; 95% CI: 0.94, 3.77). Similarly, no differences were found between donor and nondonor infants regarding feeding intolerance (vomiting) (RR: 1.26; 95% CI: 0.53, 3.01), slow weight gain (RR: 0.36; 95% CI: 0.13, 1.02), oral thrush (RR: 0.55; 95% CI: 0.12, 2.37), or need for phototherapy (RR: 2.21; 95% CI: 0.93, 5.23). The certainty of evidence was very low for all outcomes. Limited, very low certainty evidence does not support any short-term harms or benefits of human milk donation for donors or their infants. The protocols for both studies were registered with the International Prospective Register of Systematic Reviews (PROSPERO) on March 26, 2024. Study IDs: CRD42024529222 and CRD42024528803.

**Statements of significance**

This manuscript reports the results from 2 systematic reviews funded by the WHO related to the impact of human milk donation on the health of the donor and the donor’s infant. To the best of our knowledge, these are the first reviews on this topic and describe the current state of evidence on this topic.

## Introduction

The American Academy of Pediatrics, the WHO, and the European Society of Pediatric Gastroenterology, Hepatology, and Nutrition recommend the use of maternal milk as the optimal feeding source for premature/low birth weight infants, as human milk has been shown to be protective against several of the morbidity risks associated with prematurity and low birth weight [[1], [2], [3], [4], [5], [6], [7]]. When a mother’s own milk is unavailable, insufficient, or contraindicated, donor human milk (DHM) is recommended [[10], [11], [8], [9]]. The use of DHM is increasing globally, and to help meet this demand, there has been a corresponding increase in human milk banking [[12], [13], [14]]. As of 2024, human milk banks are operating in >60 countries worldwide, though most are in high-resource countries with few operating in low- and middle-income countries [[13], [14], [15]].

It is well known that DHM benefits recipient infants; however, the effects of donation on donors or their infants are not well established. To date, no systematic review has examined these potential impacts. These WHO-commissioned systematic reviews synthesize the current literature regarding the impact of human milk donation on the donor’s health and well-being and their infant’s health and growth.

## Methods

Two separate systematic reviews are summarized in this manuscript: 1 focused on health and well-being outcomes for the donor and 1 focused on health and growth outcomes for the donor’s infant. Both reviews were conducted following the standard guidelines of the Cochrane Handbook for Systematic Reviews of Interventions, and the results were reported following the PRISMA guidelines [16]. The detailed methods for these systematic reviews were published together in a comprehensive protocol [17], and the protocols were individually preregistered on the PROSPERO registry (IDs: CRD42024528803 and CRD42024529222).

### Study type

We included observational studies (cross-sectional, cohort, and before-and-after studies), quasi-experimental studies, and randomized control trials (RCTs). We excluded case reports, case series, and systematic reviews. Studies with or without control groups were included. Qualitative studies investigating the perspectives of donors on human milk donation were also excluded, as this topic has previously been reviewed (also included perspectives of recipients, healthcare professionals, and additional women) [18]. This review found altruism as a prevalent theme among donors as well as the desire for reciprocity if the donor found themselves in a situation in need of DHM and the ability for human milk donation to aid in the grieving process following the loss of a child [18].

### Study population

The study populations included lactating women (of various health statuses) and infants of lactating mothers (healthy or with various health concerns born at any gestational age and at any birth weight to lactating mothers of various health statuses) who participated in human milk donation [17].

### Exposure and comparison

The intervention of interest was human milk donation; the definition of donated human milk was adopted from the European Milk Bank Association as “breastmilk that has been expressed by a mother and provided freely to a (human milk donor bank) to be fed to another mother’s child” [14,17].

We included studies irrespective of the manner of human milk donation, that is, via a nonprofit or for-profit human milk bank, a community-based or a hospital-based human milk bank, etc. We also considered studies that involved human milk donation that occurred between family members or within various social circles without the involvement of a milk bank (milk sharing). There were no specific inclusion criteria regarding the quantity or duration of human milk donation; however, the quantity and duration of human milk donation were recorded when available. The comparison group comprised lactating individuals who did not participate in human milk donation; however, the lack of a comparison group was not an exclusion criterion.

### Outcomes: human milk donors

Donor health and nutritional outcomes of interest included the incidence of acute and chronic illness during the donation period, weight loss, BMI (in kg/m^2^), presence of abnormal BMI, and micronutrient deficiencies (iron, vitamin A, vitamin B12, vitamin D, and zinc). Abnormal BMI was defined as a BMI outside the normal range (18.5–24.9) following the initiation of human milk donation, including underweight (<18.5) and overweight/obesity (≥25.0). Underweight and overweight BMI were also analyzed separately, where reported. Psychosocial outcomes of interest included the incidence of postpartum depression, postpartum psychosis, and postpartum anxiety. Well-being outcomes of interest included the length of lactational amenorrhea, the prevention of harm (as defined by the incidence of breast cancer, ovarian cancer, postpartum hemorrhage, and type 2 diabetes), and the qualities of the lactation experience (milk supply, pumped milk feeding, breastfeeding exclusivity, breastfeeding duration, and the incidence of mastitis). Additional outcomes of interest specified after the development and publication of our protocol included the incidence of chapped or cracked nipples and the incidence of breast engorgement among human milk donors [17].

### Outcomes: infants of human milk donors

Health and nutritional outcomes of interest for infants of human milk donors included all-cause morbidity, feeding intolerance (defined as the incidence of vomiting and the incidence of diarrhea), adverse effects (growth faltering or growth failure), infections during the first year of life, micronutrient deficiencies (iron, vitamin A, vitamin B12, vitamin D, and zinc), development (further defined as Bayley scores at certain ages), and all-cause mortality. Growth outcomes of interest for infants of human milk donors included weight-for-age (*z*-score), length-for-age (*z-*score), weight-for-length (*z*-score), head circumference, and underweight, wasted, and/or stunted at certain ages. Additional outcomes of interest specified after the development and publication of our protocol included the incidence of oral thrush, the need for phototherapy due to hyperbilirubinemia, the percent of weight loss after birth, and slow weight gain.

Detailed definitions for all donor and infant outcomes are provided in Supplemental Tables 1 and 2, consistent with our published protocol [17].

### Literature search

The literature searches for both studies were conducted separately but in a systematic manner and included searches of multiple databases, including PubMed, Embase, the Cochrane Central Register for Controlled Trials, the Web of Science Core Collection, the Cumulated Index in Nursing and Allied Health Literature, Scopus, and the WHO Global Index Medicus. There were no restrictions applied to the searches based on publication date, language, or study design. The principal search strategies, developed by a medical librarian (AS), can be found in our previously published protocol [17] and can be found in Supplemental Table 3 and Supplemental Table 4. The searches also included gray literature, which entailed a search of Google Scholar and clinicaltrials.gov for ongoing studies, a search of the websites of relevant international agencies such as the WHO (including the WHO’s Reproductive Health Library), the UNICEF, the Global Alliance for Improved Nutrition, the International Food Policy Research Institute, the International Initiative for Impact Evaluation, the Nutrition International, the Human Milk Bank Association of North American, the European Milk Bank Association, and a search through citations of previously published reviews on human milk banking and the latest published studies for potentially eligible studies. The literature searches were conducted in March 2024 and updated in April 2024.

### Data extraction

The studies identified during the literature searches were exported to the software Covidence for screening [19]. Two reviewers (AB, AI) independently screened all titles and abstracts to identify relevant studies, which were then screened via full-text evaluation by 2 reviewers (AB, KB) to determine inclusion eligibility. A third reviewer (TC) resolved conflicts during the screening process. Two reviewers (AB, KB) independently completed full data extraction for each included study. The data extraction template was created prior to the literature search and can be found in the supplementary files of the published protocol [17]. Minor modifications were made to the template, primarily adjustments to account for the additional outcomes of interest specified after the publication of our protocol. Conflicts that occurred during data extraction were resolved by discussion with the assistance of the senior reviewer (AI). The following information was extracted from each study: first author, publication date, study site, study year, study population, intervention, comparison, outcomes, and risk of bias. If a study did not provide all the relevant information, we contacted the authors for further data regarding exposures and outcomes. There were 2 non-English studies [20]; Google Translate and Microsoft Bing were utilized for translation into the English language. The translated version created from both resources was compared, and any discrepancies were resolved by mutual discussion between reviewers (AB, AI).

### Risk of bias assessment

We determined the risk of bias for observational studies using the Risk of Bias in Non-randomized Studies—of Interventions scale [21]. The assessments were independently performed by 2 authors (AB and KC), and any discrepancies were resolved through discussion with the senior reviewer (AI). This tool assesses bias across 7 domains: confounding, selection of participants, classification of interventions, deviations from intended interventions, missing data, measurement of outcomes, and selection of the reported result. The judgments from each domain help determine the overall risk of bias for the outcome of interest that is being assessed. We completed this for each outcome of interest reported from a given study.

### Data synthesis

We reported the findings regarding our outcomes of interest from all included studies in a narrative synthesis and performed meta-analyses when data from multiple studies (with intervention and comparison groups) were available. The statistical software RevMan Web was utilized to complete the analyses [22]. Meta-analyses reported dichotomous outcomes with relative risk effect sizes and 95% confidence intervals (CIs). Continuous outcomes were reported with mean differences and 95% CIs. The statistical heterogeneity of effect sizes was calculated using *χ*^2^ and *I*^2^. We planned to assess small study effects using funnel plots and regression tests; however, the number of included studies in the meta-analysis was <10, so this testing was not performed.

We assessed the overall certainty of evidence for the effect of human milk donation on each outcome for the donor and the donor’s infant using the Grading of Recommendations, Assessment, Development, and Evaluation (GRADE) approach facilitated by the GradePro software [23]. We assigned certainty levels to each reported outcome as very low, low, moderate, or high. We reported the results of the GRADE assessment in the form of GRADE evidence profiles. For the impact of human milk donation on the donor, we reported GRADE evidence profiles for the following outcomes: incidence of acute illness, chronic illness, overweight BMI, postpartum depression, postpartum psychosis, postpartum anxiety, pumped breastmilk feeds, mastitis, chapped or cracked nipples, and breast engorgement. For the impact of human milk donation on the donor infant, we reported GRADE evidence profiles for the following outcomes: all-cause morbidity, feeding intolerance (vomiting), adverse effects such as slow weight gain in the first year of life per parental report, need for phototherapy due to hyperbilirubinemia, the incidence of gastrointestinal infections in the first year of life, all-cause mortality, vitamin D deficiency, and Bayley score at 2 y of age.

## Results

In the sections below, we describe the findings of our literature search and report the results for available outcomes for each respective study separately. We will first discuss the impact of human milk donation on the donor, followed by an analysis of its impact on the donor’s infant.

### The impact of human milk donation on the donor

#### Literature search

**FIGURE 1:**
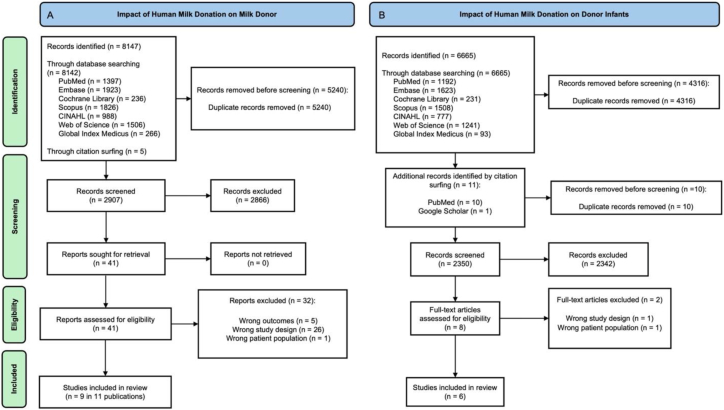
PRISMA flow diagram demonstrating the results of the literature search for both reviews. CINAHL, Cumulated Index in Nursing and Allied Health Literature; PRISMA, Preferred Reporting Items for Systematic Reviews and Meta-Analyses.

The literature search revealed 2907 titles after the exclusion of duplicates. The full text of 41 studies was screened for eligibility; 9 studies, which were available in 11 publications [20,[24], [25], [26], [27], [28], [29], [30], [31], [32], [33]], met the inclusion criteria, and 32 studies were excluded (Figure 1A). The reasoning for the exclusion of each study can be found in the supplementary information (Supplemental Table 5).

#### Characteristics of included studies

The characteristics of the included studies can be found in the supplementary information (Supplemental Table 6).

In summary, the 9 included studies were observational in design [20,[24], [25], [26], [27], [28], [29], [30], [31], [32], [33]]. Five studies were conducted in Europe (Finland [24], France [25], Italy [20], Spain [32,33], and Sweden [29, 2[29], 2 studies in North America (United States of America [30,31]), 1 study in South America; Brazil [27,28], and 1 study in Asia (India [26]). One study was published only in abstract form; we contacted the authors for more information, and they were able to provide a complete, but unpublished, nonpeer-reviewed manuscript [24]. Two studies were published in non-English languages [20,32] and were translated using machine learning from 2 different online resources, Google Translate and Microsoft Bing.

The median sample size of the included studies was 139 [24] participants, ranging from 72 [29] to 200 [20] participants. Eight studies included human milk donors who donated to a human milk bank [20,24,30,32,33], whereas 1 study included donors who shared their milk through an informal process that did not involve a designated human milk bank to serve as an intermediary [31]. Further characteristics regarding the study populations, including the method of recruitment of human milk donors, maternal age at which human milk donation occurred, reason for human milk donation, gestational age of infant, birth weight of infant, and the duration and volume of human milk donation, can be found in the supplemental information (Supplemental Table 7).

Five studies included a comparison group [26,31]. The comparison group in 2 studies consisted of human milk donation recipients [26,31], and in the remaining 3 studies, consisted of lactating mothers who were nondonors [20,24,30]. The 3 studies with comparison groups of nondonor mothers were heterogeneous; 1 study included women who had pumped milk while breastfeeding their infant over the previous 3 y but did not donate any of their pumped milk [30], whereas the other 2 studies included women who gave birth to a live infant during a similar time period but had not donated any human milk [20,24].

We contacted 5 corresponding authors for further information regarding methodology, clarification of outcomes, and data collection, with a 60% response rate [24,26,29,31].

### Effect of human milk donation on the donor’s health

#### Primary outcomes—health, nutritional, and psychosocial

##### Abnormal BMI—overweight

One study reported data on the incidence of overweight BMI in human milk donors [24]. This study included 137 total participants, with 78 in the donor group and 59 in the nondonor group. The results showed little to no difference in risk of overweight [risk ratio (RR): 1.27; 95% CI: 0.81, 2.01]; however, the wide CI indicates considerable imprecision and includes the possibility of both increased and decreased risk (Supplemental Figure 1). The certainty of evidence was very low due to study type (nonrandomized study), overall risk of bias, and imprecision of the summary estimate (Table 1). Another study reported a similar prevalence of overweight BMI among human milk donors (43%), but there was no comparison group included, and therefore, it could not be included in the meta-analysis [28].

**TABLE 1:** Grading of Recommendations, Assessment, Development, and Evaluation evidence profiles for certainty assessment of health and well-being outcomes of human milk donors Question: Does human milk donation impact the health and well-being of the human milk when compared to nonhuman milk donors?.

Certainty assessment							№ of patients		Effect		Certainty
№ of studies	Study design	Risk of bias	Inconsistency	Indirectness	Imprecision	Other considerations	Human milk donation, %	No human milk donation, %	Relative (95% CI)	Absolute (95% CI)	
Maternal acute illness during donation - not reported											
-	-	**-**	**-**	-	-	-	-	-	-	-	-
Maternal chronic illness (developed after the start of donation) - not reported											
-	-	**-**	**-**	-	-	-	-	-	-	-	-
Abnormal BMI (overweight) post initiation of donation											
1	Nonrandomized studies	Serious^1^	Not serious	Not serious	Serious^2^	None	32/78 (41.0)	19/59 (32.2)	RR: 1.27 (0.81, 2.01)	87 more per 1000 (from 61 fewer to 325 more)	⊕◯◯◯ Very low
Incidence of postpartum depression											
2	Nonrandomized studies	Serious^3^	Serious^4^	Not serious	Serious^2^	None	39/178 (21.9)	46/129 (35.7)	RR: 0.60 (0.21, 1.72)	143 fewer per 1000 (from 282 fewer to 257 more)	⊕◯◯◯ Very low
Incidence of postpartum anxiety											
2	Nonrandomized studies	Serious^3^^,^^5^	Not serious	Not serious	Serious^2^	None	59/178 (33.1)	51/129 (39.5)	RR: 0.84 (0.59, 1.18)	63 fewer per 1000 (from 162 fewer to 71 more)	⊕◯◯◯ Very low
Incidence of postpartum psychosis - not reported											
-	-	-	-	-	-	-	-	-	-	-	-
Pumped milk feeding											
2	Nonrandomized studies	Serious^6^	Serious^7^	Not serious	Serious^2^	None	167/198 (84.3)	128/170 (75.3)	RR: 1.09 (0.63, 1.89)	68 more per 1000 (from 279 fewer to 670 more)	⊕◯◯◯ Very low
Incidence of mastitis											
1	Nonrandomized studies	Serious^1^	Not serious	Not serious	Serious^2^	None	18/80 (22.5)	9/59 (15.3)	RR: 1.48 (0.71, 3.05)	73 more per 1000 (from 44 fewer to 313 more)	⊕◯◯◯ Very low
Incidence of chapped and/or cracked nipples											
1	Nonrandomized studies	Serious^6^	Not serious	Not serious	Serious^2^	None	15/80 (18.8)	18/59 (30.5)	RR: 0.61 (0.34, 1.12)	119 fewer per 1000 (from 201 fewer to 37 more)	⊕⊕◯◯ Low
Incidence of breast engorgement											
1	Nonrandomized studies	Serious^6^	Not serious	Not serious	Serious^2^	None	23/80 (28.7)	9/59 (15.3)	RR: 1.88 (0.94, 3.77)	134 more per 1000 (from 9 fewer to 423 more)	⊕◯◯◯ Very low

Abbreviations: CI, confidence interval; RR, risk ratio.

1 The study did not adjust for any of the confounders and was considered at high risk of bias.

2 The confidence around the summary estimate was wide and included a null effect.

3 The included studies did not adjust for any of the confounders. Also both the studies did not use a standardized diagnostic tool for measurement of postpartum depression and the information was self-reported.

4 The unexplained statistical heterogeneity in the pooled analysis was 87 %.

5 The included studies did not adjust for any of the confounders. Also both the studies did not use a standardized diagnostic tool for measurement of Anxiety and the information was self-reported.

6 The included studies did not adjust for any confounders.

7 Statistical heterogeneity I^2^=96 %.

##### Postpartum depression

**FIGURE 2:**
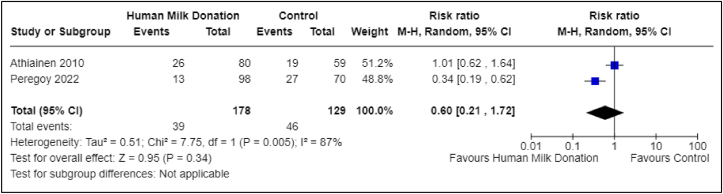
Association between human milk donation and postpartum depression. This forest plot presents the association between human milk donation and postpartum depression. CI, confidence interval.

Two studies reported on the incidence of postpartum depression [24,31]. One study included 139 total participants, with 80 in the donor group and 59 in the nondonor group [24]. The second study included 168 total participants, with 98 in the donor group and 70 in the nondonor group [31]. With the 2 studies combined, there were a total of 307 participants, with 178 in the donor group and 129 in the nondonor group. The pooled analysis suggested a possible reduction in risk (RR: 0.60; 95% CI: 0.21, 1.72); however, the CI was wide and crossed the null, indicating substantial imprecision (Figure 2). The certainty of evidence was very low due to study type (nonrandomized studies), overall risk of bias, inconsistency (the unexplained statistical heterogeneity in the pooled analysis was 87%), and imprecision of the summary estimate (wide CI around the summary estimate) (Table 1).

##### Postpartum anxiety

**FIGURE 3:**
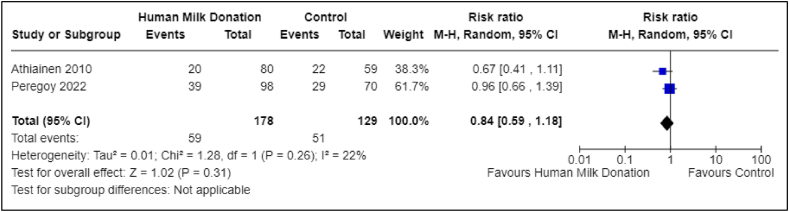
Association between human milk donation and postpartum anxiety. This forest plot presents the association between human milk donation and postpartum anxiety. CI, confidence interval.

Two studies reported on the incidence of postpartum anxiety [24,31]. One study included 139 total participants, with 80 in the donor group and 59 in the nondonor group [24]. The second study included 168 total participants, with 98 in the donor group and 70 in the nondonor group [31]. With the 2 studies combined, there were a total of 307 participants, with 178 in the donor group and 129 in the nondonor group. The pooled analysis suggested a possible lower risk to no difference in risk (RR: 0.84; 95% CI: 0.59, 1.18); the CI was relatively wide, indicating imprecision around the estimate (Figure 3). The certainty of evidence was very low due to study type (nonrandomized studies), overall risk of bias, and imprecision of the summary estimate (wide CI around the summary estimate) (Table 1). For the primary outcomes of abnormal BMI (underweight BMI and overweight BMI) and vitamin A deficiency, data were available from only the donor group, which limited further analysis. A description of these outcomes and the data available can be found in Supplemental Table 8.

None of the included studies reported on the health outcomes of illness incidence, further classified as acute illness and chronic illness, the nutritional outcomes of weight loss, BMI, iron deficiency, vitamin B12 deficiency, vitamin D deficiency, and zinc deficiency, or the psychosocial outcome of postpartum psychosis.

#### Secondary outcomes—well-being, prevention of harm, and lactation experience

##### Pumped milk feeding

Two studies reported on the use of pumping to obtain milk to feed one’s infant [20,31]. One study included 168 total participants, with 98 participants in the donor group (96% rate of pumped feeding) and 70 participants in the nondonor group (100% rate of pumped feeding) [31]. The second study included 100 participants in both the donor group and the comparison group, with 72% and 58% of the participants in each group feeding their infants pumped milk, respectively [20]. The meta-analysis suggested a minimally increased risk to no difference in risk between the 2 study groups (RR: 1.09; 95% CI: 0.63, 1.89, Supplemental Figure 2). The certainty of evidence was very low due to the study design (nonrandomized studies), overall risk of bias, inconsistency of results in the meta-analysis (*I*^2^ = 96%), and serious imprecision (wide CI around the summary estimate) (Table 1).

##### Chapped and/or cracked nipples

One study that included a comparison group reported data on the incidence of chapped or cracked nipples [24]. This study included a total of 139 participants, with 80 participants in the donor group and 59 participants in the comparison group. The analysis suggested a lower risk among donors (RR: 0.61; 95% CI: 0.34, 1.12); however, the CI crossed the null, reflecting imprecision. The certainty of evidence was very low due to the overall risk of bias and very serious imprecision (wide CI around the summary estimate) (Table 1). Three other studies without comparison groups reported the incidence of chapped or cracked nipples in human milk donors to be 6% [33], 10% [25], and 33% [30].

##### Breast engorgement

One study that included a comparison group reported data on the incidence of breast engorgement [24]. This study included a total of 139 participants, with 80 participants in the donor group and 59 participants in the nondonor group. The analysis suggested a possible increased risk among donors (RR: 1.88; 95% CI: 0.94, 3.77); however, the CI was wide and included the possibility of no effect. The certainty of the evidence was very low due to the overall risk of bias and the imprecision of the summary estimate (wide CI around the summary estimate) (Table 1). Three other studies without comparison groups reported the incidence of breast engorgement in human milk donors to be 4% [33], 19% [25], and 57% [30].

##### Mastitis

One study that included a comparison group reported data on the incidence of mastitis [24]. This study included a total of 139 participants, with 80 participants in the donor group and 59 participants in the nondonor group. The analysis suggested a possible higher risk among donors (RR: 1.48; 95% CI: 0.71, 3.05); however, the estimate was imprecise, with the CI including the possibility of no effect. The certainty of evidence was very low due to study type (nonrandomized study), overall risk of bias, and imprecision of the summary estimate (wide CI around the summary estimate) (Table 1). Three other studies without comparison groups reported the incidence of mastitis among breast milk donors to be 2% [25], 9% [33], and 20% [30].

For the secondary outcome of breastfeeding exclusivity, data were available only from the donor group, which limited further analysis. For the secondary outcome of breastfeeding duration, data were available from the donor group and nondonor group; however, further analysis was limited as the IQR was not reported. A description of these outcomes and the data available can be found in Supplemental Table 8.

None of the included studies reported on the secondary outcomes of milk supply, length of lactational amenorrhea, incidence of breast cancer, incidence of ovarian cancer, incidence of postpartum hemorrhage, or incidence of type 2 diabetes.

### The impact of human milk donation on the donor’s infant

#### Literature search

The literature search revealed 2350 titles after the exclusion of duplicates. The full text of 8 studies was screened for eligibility; 6 studies met the inclusion criteria, and 2 studies were excluded (Figure 1B). The reasoning for the exclusion of each study can be found in our supplementary information (Supplemental Table 9).

### Characteristics of included studies

The characteristics of the included studies can be found in our supplemental information (Supplemental Table 10).

All 6 included studies were observational in design [24,29,30,[32], [33], [34]]. Four studies were conducted in Europe (2 in Spain [32,33], 1 in Finland [24], and 1 in Sweden [29, 1[29], 1 study in North America (United States of America [30]), and 1 study in Asia (India [34]). One study was published in abstract form; we contacted the authors for more information, and they were able to provide a complete, but unpublished, nonpeer-reviewed manuscript [24].

The studies’ sample sizes ranged from 72 infants [29] to 415 infants [33]. All of the studies involved human milk donors who donated to human milk banks [24,29,30,[32], [33], [34]]. Further characteristics regarding the study populations, including the method of recruitment of human milk donors, maternal age at which human milk donation occurred, gestational age of donor infants, birth weight of donor infants, age of infant at initiation of human milk donation, and the duration and volume of human milk donation, can be found in our supplemental information (Supplemental Table 11). The timing of donation can be inferred from the age of the infant at initiation of human milk donation, which was reported in 5 of the 6 included studies [32,33]. The age of the infant at the initiation of human milk bank donation varied. One study reported human milk donation was initiated within 2 mo of infant delivery [24]. In 3 studies, the average age of infants at initiation of donation was reported and ranged from 9 d old (SD = 3.47 d) to 129 d old (+/‒ 135 d) [24,29,30,[32], [33], [34]]. In the final study that reported this data, they found the median age of infants at initiation of donation was 2.9 mo (IQR: 1.3‒5.7 mo) [32,33].

Two studies included a comparison group [24,30]. One study had a comparison group that consisted of nonhuman milk donors and their infants [24]. The other study had a comparison group that consisted of nonhuman milk donors who pumped to collect breast milk while nursing their infant [30].

We contacted 4 corresponding authors for further information regarding methodology, clarification of outcomes, and data collection, with a 50% response rate [24,29,33,34].

### Effect of human milk donation on the infant of the donor

#### Primary outcomes—health

##### Feeding intolerance (vomiting)

One study with a comparison group reported on feeding intolerance (vomiting) [24]. This study included a total of 139 participants, with 80 participants in the donor infant group and 59 participants in the nondonor infant group. The analysis suggested a slightly increased risk to no difference in risk (RR: 1.26; 95% CI: 0.53, 3.01); however, the CI was wide and crossed the null, reflecting imprecision. The certainty of evidence was very low due to study design (nonrandomized), overall risk of bias, and imprecision of the summary estimate (Table 2). One other study without a comparison group reported the incidence of reflux in donor infants to be 13% [30].

**TABLE 2:** Grading of Recommendations, Assessment, Development, and Evaluation evidence profiles for certainty assessment of health and growth outcomes of donor infants Question: Does human milk donation impact the health and growth of the donor infants when compared to infants of nonmilk donors?.

Certainty assessment							№ of patients		Effect		Certainty
№ of studies	Study design	Risk of bias	Inconsistency	Indirectness	Imprecision	Other considerations	Donation of human milk, %	No donation, %	Relative (95% CI)	Absolute (95% CI)	
All-cause morbidity - not reported											
-	-	-	-	-	-	-	-	-	-	-	-
Intolerance, further defined as vomiting											
1	Nonrandomized studies	Serious^1^	Not serious	Not serious	Serious^2^	None	12/80 (15.0)	7/59 (11.9)	RR: 1.26 (0.53, 3.01)	31 more per 1000 (from 56 fewer to 238 more)	⊕◯◯◯ Very low
Adverse events: slow weight gain per parenteral report											
1	Nonrandomized studies	Serious^1^	Not serious	Not serious	Serious^2^	None	5/80 (6.3)	10/59 (16.9)	RR: 0.36 (0.13, 1.02)	108 fewer per 1000 (from 147 fewer to 3 more)	⊕◯◯◯ Very low
Adverse events: presence of oral thrush											
1	Nonrandomized studies	Serious^1^	Not serious	Not serious	Very serious^2^	None	3/80 (3.8)	4/59 (6.8)	RR: 0.55 (0.12, 2.37)	31 fewer per 1000 (from 60 fewer to 93 more)	⊕◯◯◯ Very low
Adverse events: need for phototherapy due to hyperbilirubinemia											
1	Nonrandomized studies	Serious^1^	Not serious	Not serious	Serious^2^	None	18/80 (22.5)	6/59 (10.2)	RR: 2.21 (0.93, 5.23)	123 more per 1000 (from 7 fewer to 430 more)	⊕◯◯◯ Very low
Gastrointestinal infections in first year of life - not reported											
-	-	-	-	-	-	-	-	-	-	-	-
All-cause mortality											
2	Nonrandomized studies	Serious^3^	Not serious	Not serious	Not serious	None	There was no comparison group in either study. In 1 study, there were 217 donor infants with an infant mortality rate of 14.75% (*n* = 32 donor infants). In the other study, there were 415 donor infants with an infant mortality rate of 4.1% (*n* = 17). No conclusive statements could be made about the effect of the donation of human milk on infant mortality due to the lack of a comparison group.				⊕◯◯◯ Very low
Vitamin D micronutrient deficiency - not reported											
-	-	-	-	-	-	-	-	-	-	-	-
Bayle score at 2 y of age - not reported											
-	-	-	-	-	-	-	-	-	-	-	-
Growth-weight for age-not reported											
-	-	-	-	-	-	-	-	-	-		
Growth-height for age-not reported											
-	-	-	-	-	-	-	-	-	-		

Abbreviations: CI, confidence interval; RR, risk ratio.

1 The only included study was at high risk of bias because the authors did not adjust for any of the potential confounders.

2 The confidence interval around the summary estimate was wide and included a null effect.

3 Both included studies did not have a comparison group, so the true association of mortality with the donation of human milk cannot be determined.

##### Adverse effects, such as growth faltering or growth failure

One study reported on adverse effects such as growth faltering or growth failure [24]. This study included a total of 139 participants, with 80 participants in the donor infant group and 59 in the nondonor infant group. The results suggested a potential reduction in risk among donor infants (RR: 0.36; 95% CI: 0.13, 1.02); however, the CI was wide and crossed the null, indicating imprecision. The certainty of evidence was very low due to study design (nonrandomized), overall risk of bias, and imprecision of the summary estimate (Table 2).

##### All-cause mortality

Two studies without comparison groups reported on all-cause mortality [33,34]. One study included a total of 217 donor infants with an infant mortality rate of 14.75% [34]. The other study included a total of 415 donor infants with an infant mortality rate of 4.1% [33].

For the primary outcome of feeding intolerance (diarrhea), no studies formally reported data; however, 1 participant spontaneously reported this concern in 1 study, which is further detailed in Supplemental Table 12.

None of the included studies reported on the health outcomes of all-cause morbidity (although 1 study did investigate barriers to donation with 1 donor sharing that a barrier they experienced was their child falling ill without further explanation [24]), infections during the first year of life including gastrointestinal, respiratory, central nervous system or other, or micronutrient deficiencies such as iron, vitamin A, vitamin B12, vitamin D, and zinc.

#### Secondary outcomes—growth, development, and additional outcomes

##### Oral thrush

One study with data available for the comparison group reported on the additional outcome of interest of oral thrush [24]. This study included a total of 139 infants, with 80 participants in the donor infant group and 59 in the nondonor infant group. The analysis suggested a lower risk among donor infants (RR: 0.55; 95% CI: 0.12, 2.37); however, the CI was very wide and crossed the null, reflecting substantial imprecision. The certainty of evidence was very low due to study design (nonrandomized), overall risk of bias, and imprecision of the summary estimate (Table 2). Another study without data for the comparison group reported on this outcome [30]. This study had a total of 106 infants, with 87 in the donor infant group and 19 in the nondonor infant group. In the donor infant group, 11 of the 87 infants experienced oral thrush; the authors reported that there was no significant difference between the donor infant group and the nondonor infant group.

##### Need for phototherapy due to hyperbilirubinemia

One study reported on the additional outcome of interest need for phototherapy due to hyperbilirubinemia [24]. This study included a total of 139 infants, with 80 participants in the donor infant group and 59 in the nondonor infant group. The analysis suggested a possibly higher risk among donor infants (RR: 2.21; 95% CI: 0.93, 5.23); however, the CI was wide and included the possibility of no effect. The certainty of evidence was very low due to study design (nonrandomized), overall risk of bias, and imprecision of the summary estimate (Table 2).

The additional outcome of percent weight loss after birth is detailed further in Supplemental Table 12.

None of the included studies reported on the developmental outcomes of the Bayley score at specified ages or the growth outcomes pertaining to weight, length, head circumference, and the presence of underweight, wasting, and/or stunting at certain ages.

### Additional analyses

For both systematic reviews, to measure the impact of human milk donation on the donor and the donor’s infant, we planned to conduct subgroup analyses based on the phase of milk donation (colostrum, transitional, and mature milk), presence of bereavement (donation following perinatal death compared with donation after live birth), whether donation occurred after full-term or preterm birth, and whether donation occurred after birth of an infant(s) with normal birth weight compared with low birth weight. No studies were identified that reported data for these subgroups, so no subgroup analyses were conducted. Additionally, we had planned to conduct sensitivity analyses comparing fixed and random effect models, as well as analyses focusing on studies with a high risk of bias. However, due to the limited number of studies, these sensitivity analyses could not be performed.

## Discussion

### Summary of main results

The 2 systematic reviews included in this manuscript evaluated the effect of human milk donation on the health and well-being of the donor and the health and growth of the donor’s infant. The available data demonstrated, with very low certainty evidence, that there is no association between human milk donation and increased incidence of overweight BMI, postpartum depression, postpartum anxiety, pumped milk feeding, mastitis, chapped and/or cracked nipples, or breast engorgement in donors and that there is no association between human milk donation and increased incidence of feeding intolerance (vomiting), slow weight gain, need for phototherapy due to hyperbilirubinemia, and oral thrush in infants of donors.

### Completeness and applicability of results

Although 1 study suggested a protective effect of human milk donation on the incidence of postpartum depression among breast milk donors, when meta-analyzed with a second study, the association was no longer apparent (RR: 0.60; 95% CI: 0.21, 1.72), and the wide CI reflects substantial imprecision (Figure 2). A protective effect of donation against postpartum depression is biologically plausible, as breastfeeding in general is associated with a lower risk of postpartum depression [35]. However, our analysis did not show a positive or negative association between human milk donation and postpartum depression. Increased milk production as a result of milk donation could be hypothesized to lead to adverse effects such as breast engorgement and mastitis. However, our systematic review and meta-analysis did not identify any difference between the donor and nondonor groups regarding the incidence of these adverse events. As the certainty of evidence was very low for all outcomes in this review, all observations should be considered preliminary and tested in future studies with rigorous designs.

Two studies reported on the infant mortality rate in infants of human milk donors [33,34]. In 1 study, the infant mortality rate was 4.1% (17/415 donor infants); the infant deaths were not related to the amount of milk donated [33]. This study was conducted in Spain, and these infants were admitted to the neonatal intensive care unit (NICU) and died during their hospitalization. This reported mortality rate is significantly higher than the country’s infant mortality rate of 3 deaths per 1000 live births and the country’s neonatal mortality rate of 2 deaths per 1000 live births [36]. The authors were contacted for further information, but we did not receive a response. In the other study, the infant mortality rate was 14.75% (32/217 donor infants) [34]. This study was conducted in India; the country’s infant mortality rate is 26 deaths per 1000 live births, and the country’s neonatal mortality rate is 18 deaths per 1000 live births [37]. The authors were contacted for further information, but we did not receive a response. Neither of these studies included comparison groups, so it is not possible to compare these high mortality rates to the mortality rates of infants of nondonors in the same geographic area and during the same time period. We suspect these high mortality rates are due to small sample sizes and selection bias, given that many infants included in the studies had medical needs requiring extended NICU hospitalization. Additionally, in the latter study, most infants were small for gestational age at birth and most demonstrated low birth weight, both of which are independent risk factors for infant decline and death [38,39]. Overall, the current evidence does not support any association between human milk donation and increased risk of infant mortality among donor infants.

One study reported data on slow weight gain [24], and the analysis suggested a reduced risk of slow weight gain among donor infants (RR: 0.36; 95% CI: 0.13, 1.02); however, the wide CI crossed the null, indicating imprecision and very low certainty of evidence. One hypothesis for a seemingly protective effect of donation against slow weight gain could be the increased likelihood of healthy mothers with sufficient milk production choosing to donate, and therefore an increased likelihood that their infants were born full-term and at a healthy birth weight. However, the evidence is of very low certainty, and therefore, no conclusion can be drawn from this data.

An important contextual factor is the reasoning behind the lactating woman’s decision to engage in pumping. The reasoning may involve or include a prior NICU stay, return to work, bereavement, or maternal preference. These underlying circumstances could independently influence maternal psychological well-being, lactation complications, and infant outcomes, yet were not consistently reported across studies. In addition, unmeasured confounding variables such as maternal socioeconomic status, psychosocial support, and access to lactation services were not fully accounted for in the included studies, which further limits the certainty of the evidence.

### Quality of evidence

Both systematic reviews summarized evidence from a small number of observational studies. Nine observational studies were included to investigate the impact of human milk donation on the health and well-being of the donor [20,[24], [25], [26], [27],[29], [30], [31], [32]] and 6 observational studies were included to investigate the impact of human milk donation on the health and growth of the donor’s infant [24,29,30,[32], [33], [34]], all of which had an overall high risk of bias primarily due to inadequate adjustment for confounding variables. This limitation reflects the inherent challenge of observational research and adjusting data; if an adjusted estimate had been available, this would have been extracted for use in this review. Further, studies investigating outcomes such as postpartum depression and postpartum anxiety did not utilize any of the available standardized diagnostic tools capable of providing an objective measure. Both systematic reviews included studies that lacked a comparison group, precluding the possibility of drawing any conclusions about the risks and benefits of donation. Among the studies with both intervention and comparison groups, the summary estimates were imprecise, even in those that underwent meta-analysis. All study outcomes had very low certainty of evidence.

### Potential bias in the review process

We followed the standard guidelines of the Cochrane Collaboration to conduct both systematic reviews. We adopted a broad search strategy, utilized multiple databases, and examined 2907 titles and abstracts for the impact of human milk donation on the donor and 2350 titles and abstracts for the impact of human milk donation on the donor infant, which included published studies and nonpublished data from ongoing and past studies. We performed our analysis based on our previously published protocol [17], with the exception of incorporating outcomes of breast engorgement and chapped/cracked nipples, post hoc. These additional outcomes were included after mutual discussion among authors, as we determined they were clinically relevant and would enhance the comprehensiveness of our findings. Additionally, we prespecified the outcomes to include in the GRADE profile tables for both studies; however, we expanded the outcomes included in the tables as there was minimal to no data collected for many of the prespecified outcomes, but there was data available for additional outcomes.

### Limitations of the systematic review

There were multiple limitations to both systematic reviews. For both, the number of included studies was small, the studies included were all observational (there was an absence of high-quality studies, including a lack of RCTs), and data were not available for most of the outcomes specified in our previously published protocol [17]. We were therefore unable to perform any analyses for publication or reporting biases. We acknowledge that there was substantial heterogeneity in the included studies. In addition to clinical heterogeneity related to differences in study populations, exposures, and outcomes, there was methodological heterogeneity, including variability in study design, outcome definitions, duration of follow-up, and risk of bias. There was also statistical heterogeneity, reflected by variability in effect sizes and wide CIs. Given these limitations, it could be argued that meta-analysis may not have been appropriate for some outcomes. Nevertheless, we chose to present pooled estimates alongside detailed descriptions in figures, tables, and text to allow readers to assess both individual study findings and the overall evidence.

Another limitation is the variability in outcome measurement across studies. For example, postpartum depression and anxiety were assessed using different screening tools or via self-reported data, and complications of lactation, such as mastitis or engorgement, were variably defined, which introduces measurement variance. In addition, many maternal outcomes relied on self-reported recall, which is subject to recall bias. Finally, an important contextual factor is whether pumping was a maternal choice or a necessity (e.g., due to a NICU stay, return to work, or bereavement). This distinction may influence maternal psychological well-being and infant feeding outcomes, but it was not consistently addressed in the included studies.

Furthermore, these reviews do not address the emotional and psychological harms or benefits of human milk donation for the donor, as a previously published review focused specifically on this topic [18]. That review highlighted that motivations for donation often include altruism, a desire for reciprocity if the need arises, and, for some mothers, a means of coping with grief after perinatal loss. At the same time, donors may also experience psychological challenges, such as stress related to time commitments or emotional burden. These psychosocial dimensions are important to acknowledge, as they may influence maternal well-being, breastfeeding practices, and ultimately the outcomes of interest in this review [18]. Future research integrating psychosocial outcomes with clinical outcomes would provide a more comprehensive understanding of the impact of human milk donation.

Another important consideration is the inclusion of 1 study on milk sharing [31]. Although most human milk donation occurs through milk banks, there are also reports of milk being shared directly between individuals outside of milk banks [40]. In these contexts, the donor typically knows the recipient, which may influence motivations, emotional experiences, and psychosocial outcomes differently than anonymous donation through human milk banks. Although this study does not fully align with the other included studies, it provides an important representation of real-world practices. Its inclusion highlights the diversity of milk donation experiences, but it also limits direct comparability across studies.

### Comparison to previously published literature

To the best of our knowledge, there are no prior published systematic reviews or meta-analyses exploring the impact of human milk donation on the health and well-being of the donor or on the health and growth of the donor’s infant. Our systematic reviews and meta-analyses add to the literature a concise outline of the current state of research regarding the effect of human milk donation on the health and well-being of donors and the health and growth of donor infants.

### Implications for practice

The implication for clinical practice from both systematic reviews is that there is no significant evidence to support or oppose the practice of human milk donation in terms of its relation to the donor’s health and well-being or the health and growth of the donor’s infant.

### Implications for research

There are many implications for research from both systematic reviews. The inclusion of 9 and 6 studies, respectively, demonstrates the relative paucity of research investigating this topic. Overall, more studies investigating this topic are needed. Furthermore, all of the included studies were observational, and 4 studies in each review did not have comparison groups [25,27,29,[32], [33], [34]]. The feasibility of an RCT investigating this topic is ethically challenging; however, observational studies with robust design (i.e., establishing eligibility criteria, matching participants, utilizing random sampling, blinding, etc.) may be more feasible and provide useful evidence. Future studies should collect detailed information on the timing of human milk donation (colostrum, transitional, or mature milk), duration of donation (in days, weeks, etc.), and amount of donation (total volume donated and/or number of donations made), along with long-term follow-up to establish temporality between exposure and outcomes. Future studies should also assess for any differential effects based on factors such as donation after loss of a child compared with donation after live birth, donation after preterm birth compared with full-term birth, and donation after delivery of a low birth weight infant compared with a normal birth weight infant. Additionally, future studies should investigate human milk donation and human milk banking in low-resource settings, as 7 of the 9 included studies investigating the impact of donation on donors were conducted in high-income countries [20,24,25,[29], [30], [31], [32]] and 5 of the 6 included studies investigating the impact of donation on donor infants were conducted in developed countries [24,29,30,32,33]. Maternal and infant malnutrition is prevalent in low- and middle-income countries, and human milk donation in these settings might have more consequential effects on donor infants [13].

In conclusion, the available evidence does not demonstrate any short-term harm or benefit of human milk donation on the health and well-being of the donor or the health and growth of the donor infant; however, the body of evidence is limited and of low certainty. Future studies with robust designs are needed to investigate the effect of human milk donation on the health and well-being of the donor and the health and growth of the donor infant.

## Author contributions

The authors’ responsibilities were as follows—KEB, AB: contributed to the design of the data collection instruments, data collection, the writing of the initial manuscript draft, and critically reviewed and revised the manuscript; TC: contributed to the initial conceptualization of the study and critically reviewed and revised the manuscript; AS and JE: designed and executed the search strategy; JR: contributed to data extraction, the writing of the manuscript, and critically reviewed and revised the manuscript; MHM and ZAB: contributed to the design of the study, the development and the interpretation of the Grading of Recommendations, Assessment, Development, and Evaluation (GRADE) analysis, and critically reviewed and revised the manuscript; AI: conceptualized and designed the study, contributed to screening of titles and abstracts, helped with the design of the data collection sheet, conducted the meta-analyses and the GRADE analyses, contributed to the initial manuscript draft, critically reviewed and revised the manuscript, and he is the guarantor of this work. All authors agree to be accountable for all aspects of the work; and all authors: read and approved the final manuscript.

## Data availability

Data are available upon request.

## Funding

WHO [performance of work (APW), grant/award number: 2024/1445457]. The findings from this review were reviewed by the guideline development group at WHO; however, this manuscript does not endorse or represent the official position of WHO on this matter. Authors are responsible for the accuracy of the content and interpretation of the data.

## Declaration of AI and AI-Assisted Technologies in the Writing Process

During the preparation of this work, the author(s) used Google Translate and Microsoft Bing in order to translate studies that were originally in a non-English language. There was no other use of AI or AI-assisted technologies in the preparation of this manuscript. After using this tool/service, the author(s) reviewed and edited the content as needed and take(s) full responsibility for the content of the publication.

## Conflict of interest

ZAB is an editorial board member for Advances in Nutrition and played no role in the journal’s evaluation of the manuscript. All other authors report no conflicts of interest.
