# Supplementary material for: The Impact of Donating Human Milk on the Health of the Donor and Their Infant: Evidence from Two Systematic Reviews
Source: Adv Nutr. 2025 Dec 30;17(2):100581. doi: 10.1016/j.advnut.2025.100581 (PMC12861158; doi:10.1016/j.advnut.2025.100581)
Supplement: multimedia component 1 [file mmc1.pdf]

Supplementary Document

The impact of donating human milk on the health of the donor and their infant: Evidence from two systematic reviews

Kendall E. Baier[1], Alaina Berg[1], Abigail Smith[2], James Evans[2], Jaimie Rogner[3], Mohammed H. Murad[4], Tarah Colaizy[5], Zulfiqar A. Bhutta[6,7,8], and Aamer Imdad[9]*

[1] Stead Family Department of Pediatrics, University of Iowa Carver College of Medicine, Iowa City, IA, USA [2] Health Sciences Library, State University of New York Upstate Medical University, Syracuse, NY, USA, [3] NewYork-Presbyterian Hospital/Weill Cornell Medical Center, New York, NY, [4] Department of Internal Medicine, Division of Public Health, Infectious Diseases and Occupational Medicine, Mayo Clinic, Rochester, MN, [5] Stead Family Department of Pediatrics, Division of Neonatology, University of Iowa Carver College of Medicine, Iowa City, IA, USA, [6] Centre for Global Child Health, Hospital for Sick Children, Toronto, ON, CAN, [7] Institute for Global Health and Development, Aga Khan University, Karachi, Pakistan, [8] Department of Nutrition, Joannah and Brian Lawson Centre for Child Nutrition, University of Toronto, Toronto, ON, CAN, [9] Stead Family Department of Pediatrics, Division of Gastroenterology, Hepatology, Pancreatology, and Nutrition, University of Iowa, Iowa City, IA, USA

Contents

Table S1. Defined outcomes of interest for the impact of human milk donation on the donor. . 2

Table S2. Defined outcomes of interest for the impact of human milk donation on the donor's infant. 3

Table S3. The impact of human milk donation on the donor: Search strategies by database; search conducted on April 28th, 2024........ 4

Table S4. The impact of human milk donation on the donor infant: Search strategies by database; search conducted on April 28th, 2024. 6

Table S5. The impact of human milk donation on the donor: Table of excluded studies. 8

Table S6. The impact of human milk donation on the donor: Characteristics of included studies including population, exposure, comparison, and outcomes........ 12

Figure S1: Risk of Abnormal BMI - Overweight. This forest plot demonstrates the risk of having an overweight BMI associated with human milk donation........ 19

Table S8. The impact of human milk donation on the donor: Outcomes of interest from studies lacking information necessary for further analysis. 19

Figure S2: Risk of Pumped Milk Feeding. This forest plot demonstrates the risk of needing to feed one's own infant pumped milk associated with human milk donation. 20

Table S9. The impact of human milk donation on the donor's infant: Table of excluded studies........ 20

Table S10. The impact of human milk donation on the donor infant: Characteristics of included studies including population, exposure, comparison, and outcomes........ 20

Table S12. The impact of human milk donation on the donor infant: Further outcomes of interest........ 24

Table S1. Defined outcomes of interest for the impact of human milk donation on the donor.

Outcome		Definition
**Health Outcomes**	Acute Illness	The incidence of any illness requiring an acute visit to a healthcare facility (inpatient or outpatient care) during the donation period; episodes per duration of follow-up.
	Chronic Illness	The incidence of a condition lasting at least a year, which either limits daily activity and/or requires continuous medical attention.
**Nutritional Outcomes**	Weight Loss	The amount of weight lost at 6 months following the initiation of human milk donation.
	BMI	A BMI measured at 6 months following the initiation of human milk donation.
	Abnormal BMI	An abnormal BMI measured at 6 months following the initiation of human milk donation.
	Micronutrient Deficiencies	The incidence of iron deficiency based on ferritin and hemoglobin level, vitamin A deficiency based on serum retinol levels, vitamin B12 deficiency based on serological markers, vitamin D deficiency based on serum 25-OH-vitamin D3 level, and zinc deficiency based on serum zinc level.
**Psychosocial Outcomes**	Postpartum Depression	The incidence of postpartum depression as defined by an experience of the following symptoms for > 2 weeks: change in sleep, interest, guilt, energy level, mood, concentration, anxiety, appetite, and/or thoughts of hurting oneself or others.
	Postpartum Psychosis	The incidence of postpartum psychosis as defined by an experience of psychotic symptoms in the postpartum period, including delusions or hallucinations.
	Postpartum Anxiety	The incidence of postpartum anxiety defined by an experience of anxiety symptoms at any point postpartum or during human milk donation.
**Well-being and Prevention of Harm Outcomes**	Length of Lactational Amenorrhea	The length of lactational amenorrhea in days following delivery of their infant while lactating.
	Breast Cancer	The incidence of breast cancer development following human milk donation at the longest follow-up visit.
	Ovarian Cancer	The incidence of ovarian cancer development following human milk donation at the longest follow-up visit.
	Postpartum Hemorrhage	The incidence of postpartum hemorrhage as reported subjectively or via clinical records.
	Type 2 Diabetes	The incidence of type 2 diabetes following human milk donation at the longest follow-up visit.

**Lactation Experience Outcomes**	Milk Supply	The donor's average breast milk supply as defined in milliliters per day.
	Pumped Milk Feeding	The need to pump breast milk to feed their own infant, or lack thereof.
	Breastfeeding Exclusivity	The decision to exclusively breastfeed one's infant during donation, or lack thereof, for the first 6 months of the infant's life.
	Breastfeeding Duration	The length of time the donor participated in breastfeeding for their own infant and produced milk for donation.
	Mastitis	The incidence of symptoms aligning with mastitis per subjective report or clinical diagnosis.
**Additional Outcomes – specified after the publication of our protocol.[1]**	Chapped or Cracked Nipples	The incidence of chapped skin or cracked skin of the nipple or surrounding areola per subjective report.
	Breast Engorgement	The incidence of feeling full in the breast per subjective report.

Table S2. Defined outcomes of interest for the impact of human milk donation on the donor's infant.

**Outcome**		**Definition**
**Health Outcomes**	All-cause Morbidity	The number of acute illnesses requiring a visit to a healthcare facility as an inpatient or an outpatient during the donation period; episodes per duration of follow-up.
	Feeding Intolerance – Vomiting	The incidence of vomiting in the infant during the donation period.
	Feeding Intolerance – Diarrhea	The incidence of diarrhea in the infant during the donation period.
	Adverse Effects - Growth	The incidence of growth faltering or failure to thrive during the first year of life.
	Infections – Gastrointestinal	The number of gastrointestinal infections during the first year of life; measured by illness episodes.
	Infections – Respiratory	The number of respiratory infections during the first year of life; measured by illness episodes.
	Infections – Other	The number of infections excluding gastrointestinal or respiratory during the first year of life; this includes central nervous system infections, measured by illness episodes.
	Micronutrient Deficiencies	The incidence of iron deficiency based on ferritin and hemoglobin level, vitamin A deficiency based on serum retinol levels, vitamin B12 deficiency based on serological markers, vitamin D deficiency based on

		serum 25-OH-vitamin D3 level, and zinc deficiency based on serum zinc level.
	All-cause Mortality	The death of the infant due to any cause before the age of 1 year.
	Bayley Score (Age 2)	The score the child receives from the Bayley Scale at 2 years of age.
**Growth Outcomes**	Weight-for-Age	The z-score of weight-for-age at 6 months and 12 months of age.
	Length-for-Age	The z-score of length-for-age at 6 months and 12 months of age.
	Weight-for-Length	The z-score of weight-for-length at 6 months and 12 months of age.
	Head Circumference	The child's head circumference at 1 year of age.
	Underweight	The incidence of weight-for-age z-score less than -2 at 1 year of age.
	Stunted	The incidence of height-for-age z-score less than -2 at 1 year of age.
	Wasted	The incidence of weight-for-length z-score less than -2 at 1 year of age.
**Additional Outcomes – specified following publication of protocol.**	Oral Thrush	The incidence of signs or symptoms consistent with oral thrush during or following donation.
	Need for Phototherapy due to Hyperbilirubinemia	The incidence of phototherapy treatment for the infant due to hyperbilirubinemia during or following donation.
	Weight Loss After Birth	The percent weight loss the infant experienced after birth.
	Slow Weight Gain	The incidence of parental report of slow weight gain following birth during donation or after donation occurred.

Table S3. The impact of human milk donation on the donor: Search strategies by database; search conducted on April 28th, 2024.

Database	Search Strategies
PubMed	(("Milk Banks"[MeSH Terms] OR ("milk bank*"[Title/Abstract] OR "breastmilk bank*"[Title/Abstract])) AND ("donor*"[Title/Abstract] OR "donat*"[Title/Abstract])) OR ("breastmilk donor*"[Title/Abstract] OR "milk donat*"[Title/Abstract] OR "breastmilk donat*"[Title/Abstract] OR "breastmilk shar*"[Title/Abstract] OR "milk shar*"[Title/Abstract] OR "donor human milk"[Title/Abstract] OR "donor breast milk"[Title/Abstract] OR "donor breastmilk"[Title/Abstract] OR "pasteurized human milk"[Title/Abstract] OR "banked milk"[Title/Abstract] OR "donor milk"[Title/Abstract])
Embase	#7 #5 OR #6<br>#6 #1 AND #2

	#5 #3 OR #4<br>#4 'breastmilk donor*':ti,ab OR 'milk donat*':ti,ab OR 'breastmilk donat*':ti,ab OR 'breastmilk shar*':ti,ab OR 'milk shar*':ti,ab OR 'donor human milk':ti,ab OR 'donor breast milk':ti,ab OR 'donor breastmilk':ti,ab OR 'pasteurized human milk':ti,ab OR 'banked milk':ti,ab OR 'donor milk':ti,ab<br>#3 'donor milk'/exp<br>#2 'donor'/exp OR 'donor*':ti,ab OR 'donat*':ti,ab<br>#1 'milk bank'/exp OR 'milk bank*':ti,ab OR 'breastmilk bank*':ti,ab
Cochrane Library (CENTRAL; Cochrane Database of Systematic Reviews)	#1 Mesh descriptor: [milk banks] explode all trees<br>#2 (milk NEXT bank*):ti,ab OR (breastmilk NEXT bank*):ti,ab<br>#3 #1 OR #2<br>#4 donor:ti,ab OR donat*:ti,ab<br>#5 #3 AND #4<br>#6 (breastmilk NEXT donor*):ti,ab OR (milk NEXT donat*):ti,ab OR (breastmilk NEXT donat*):ti,ab OR (breastmilk NEXT shar*):ti,ab OR (milk NEXT shar*):ti,ab OR (donor NEXT human NEXT milk):ti,ab OR (donor NEXT breast NEXT milk):ti,ab OR (donor NEXT breastmilk):ti,ab OR (pasteurized NEXT human NEXT milk):ti,ab OR (banked NEXT milk):ti,ab OR (donor NEXT milk):ti,ab<br>#7 #5 OR #6
Scopus	(TITLE-ABS-KEY ("milk bank*" OR "breastmilk bank*") AND TITLE-ABS-KEY ("donor*" OR "donat*")) OR TITLE-ABS-KEY ("breastmilk donor*" OR "milk donat*" OR "breastmilk donat*" OR "breastmilk shar*" OR "milk shar*" OR {donor human milk} OR {donor breast milk} OR {donor breastmilk} OR {pasteurized human milk} OR {banked milk} OR {donor milk})
CINAHL	S13 S8 OR S12<br>S12 S9 OR S10 OR S11<br>S11 AB ("breastmilk donor*" OR "milk donat*" OR "breastmilk donat*" OR "breastmilk shar*" OR "milk shar*" OR "donor human milk" OR "donor breast milk" OR "donor breastmilk" OR "pasteurized human milk" OR "banked milk" OR "donor milk")<br>S10 TI ("breastmilk donor*" OR "milk donat*" OR "breastmilk donat*" OR "breastmilk shar*" OR "milk shar*" OR "donor human milk" OR "donor breast milk" OR "donor breastmilk" OR "pasteurized human milk" OR "banked milk" OR "donor milk")<br>S9 (MH "Donor Milk")<br>S8 S4 AND S7<br>S7 S5 OR S6<br>S6 AB ("donor*" OR "donat*")<br>S5 TI ("donor*" OR "donat*")<br>S4 S1 OR S2 OR S3<br>S3 AB ("milk bank*" OR "breastmilk bank*")<br>S2 TI ("milk bank*" OR "breastmilk bank*")<br>S1 (MH "Milk Banks")
Web of Science Core Collection	5 #3 OR #4

(SCI-Expanded; SSCI; ESCI; CPCI-S)	4 TS=("breastmilk donor*" OR "milk donat*" OR "breastmilk donat*" OR "breastmilk shar*" OR "milk shar*" OR "donor milk" OR "donor human milk" OR "donor breast milk" OR "donor breastmilk" OR "pasteurized human milk" OR "banked milk")<br>3 #2 AND #1<br>2 TS=(donor* OR donat*)<br>1 TS=("milk bank*" OR "breastmilk bank*")
Global Index Medicus (AIM; IMEMR; IMSEAR; LILACS; WPRO)	(mh:("Milk Banks")) OR (ti:("milk bank" OR "milk banks" OR "milk banking" OR "breastmilk bank" OR "breastmilk banks" OR "breastmilk banking") OR (ab:("milk bank" OR "milk banks" OR "milk banking" OR "breastmilk bank" OR "breastmilk banks" OR "breastmilk banking"))) AND ((ti:(donor*)) OR (ab:(donor*)) OR ((ti:(donat*)) OR (ab:(donat*)))) OR (ti:("milk donation" OR "milk donations" OR “milk sharing" OR "donor milk" OR “donor human milk” OR “donor breast milk” OR “donor breastmilk" OR “pasteurized human milk”) OR (ab:("milk donation" OR "milk donations" OR “milk sharing" OR "donor milk" OR “donor human milk” OR “donor breast milk” OR “donor breastmilk" OR “pasteurized human milk”)))

Table S4. The impact of human milk donation on the donor infant: Search strategies by database; search conducted on April 28th, 2024.

Database	Search Strategies
PubMed	((("Milk Banks"[MeSH Terms] OR ("milk bank*"[Title/Abstract] OR "breastmilk bank*"[Title/Abstract])) AND ("donor*"[Title/Abstract] OR "donat*"[Title/Abstract])) OR ("breastmilk donor*"[Title/Abstract] OR "milk donat*"[Title/Abstract] OR "breastmilk donat*"[Title/Abstract] OR "breastmilk shar*"[Title/Abstract] OR "milk shar*"[Title/Abstract] OR "donor milk"[Title/Abstract] OR "donor human milk"[Title/Abstract] OR "donor breast milk"[Title/Abstract] OR "donor breastmilk"[Title/Abstract] OR "pasteurized human milk"[Title/Abstract] OR "banked milk"[Title/Abstract])) AND ("Infant"[MeSH Terms] OR "infant*"[Title/Abstract] OR "infancy"[Title/Abstract] OR "baby"[Title/Abstract] OR "babies"[Title/Abstract] OR "neonat*"[Title/Abstract] OR "neo nat*"[Title/Abstract] OR "newborn*"[Title/Abstract] OR "new born*"[Title/Abstract] OR "newly born*"[Title/Abstract])
Embase	#11 #7 AND #10<br>#10 #8 OR #9<br>#9 'infant*':ti,ab OR 'infancy':ti,ab OR 'baby':ti,ab OR 'babies':ti,ab OR 'neonat*':ti,ab OR 'neo nat*':ti,ab OR 'newborn*':ti,ab OR 'new born*':ti,ab OR 'newly born*':ti,ab<br>#8 'infant'/exp<br>#7 #5 OR #6<br>#6 #1 AND #2<br>#5 #3 OR #4<br>#4 'breastmilk donor*':ti,ab OR 'milk donat*':ti,ab OR 'breastmilk donat*':ti,ab OR 'breastmilk shar*':ti,ab OR 'milk shar*':ti,ab OR 'donor human milk':ti,ab OR 'donor breast milk':ti,ab OR 'donor breastmilk':ti,ab OR 'pasteurized human milk':ti,ab OR 'banked milk':ti,ab OR 'donor milk':ti,ab

	#3 'donor milk'/exp<br>#2 'donor'/exp OR 'donor*':ti,ab OR 'donat*':ti,ab<br>#1 'milk bank'/exp OR 'milk bank*':ti,ab OR 'breastmilk bank*':ti,ab
Coachrane Library (CENTRAL; Cochrane Database of Systematic Reviews)	#1 Mesh descriptor: [milk banks] explode all trees<br>#2 (milk NEXT bank*):ti,ab OR (breastmilk NEXT bank*):ti,ab<br>#3 #1 OR #2<br>#4 donor*:ti,ab OR donat*:ti,ab<br>#5 #3 AND #4<br>#6 (breastmilk NEXT donor*):ti,ab OR (milk NEXT donat*):ti,ab OR (breastmilk NEXT donat*):ti,ab OR (breastmilk NEXT shar*):ti,ab OR (milk NEXT shar*):ti,ab OR (donor NEXT human NEXT milk):ti,ab OR (donor NEXT breast NEXT milk):ti,ab OR (donor NEXT breastmilk):ti,ab OR (pasteurized NEXT human NEXT milk):ti,ab OR (banked NEXT milk):ti,ab OR (donor NEXT milk):ti,ab<br>#7 #5 OR #6<br>#8 MeSH descriptor: [Infant] explode all trees<br>#9 infant*:ti,ab OR infancy:ti,ab OR baby:ti,ab OR babies:ti,ab OR neonat*:ti,ab OR (neo NEXT nat*):ti,ab OR newborn*:ti,ab OR (new NEXT born*):ti,ab OR (newly NEXT born*):ti,ab<br>#10 #8 OR #9<br>#11 #7 AND #10
Scopus	((TITLE-ABS-KEY ("milk bank*" OR "breastmilk bank*") AND TITLE-ABS-KEY (donor* OR donat*)) OR TITLE-ABS-KEY ("breastmilk donor*" OR "milk donat*" OR "breastmilk donat*" OR "breastmilk shar*" OR "milk shar*" OR {donor human milk} OR {donor breast milk} OR {donor breastmilk} OR {pasteurized human milk} OR {banked milk} OR {donor milk})) AND (TITLE-ABS-KEY(infant* OR infancy OR baby OR babies OR neonat* OR "neo nat*" OR newborn* OR "new born*" OR "newly born*"))
CINAHL	S18 S13 AND S17<br>S17 S14 OR S15 OR S16<br>S16 AB ("infant*" OR "infancy" OR "baby" OR "babies" OR "neonat*" OR "neo nat*" OR "newborn*" OR "new born*" OR "newly born*")<br>S15 TI ("infant*" OR "infancy" OR "baby" OR "babies" OR "neonat*" OR "neo nat*" OR "newborn*" OR "new born*" OR "newly born*")<br>S14 (MH "Infant+")<br>S13 S8 OR S12<br>S12 S9 OR S10 OR S11<br>S11 AB (“breastmilk donor*” OR “milk donat*” OR “breastmilk donat*” OR “breastmilk shar*” OR “milk shar*" OR “donor milk” OR “donor human milk” OR “donor breast milk” OR “donor breastmilk” OR “pasteurized human milk” OR “banked milk” OR “donor milk”)<br>S10 TI (“breastmilk donor*” OR “milk donat*” OR “breastmilk donat*” OR “breastmilk shar*” OR “milk shar*” OR “donor milk” OR “donor human milk” OR “donor breast milk” OR “donor breastmilk” OR “pasteurized human milk” OR “banked milk” OR “donor milk”)<br>S9 (MH “Donor Milk”)

	S8 S4 AND S7<br>S7 S5 OR S6<br>S6 AB (“donor*” OR “donat*”)<br>S5 TI (“donor*” OR “donat*”)<br>S4 S1 OR S2 OR S3<br>S3 AB (“milk bank*” OR “breastmilk bank*”)<br>S2 TI (“milk bank*” OR “breastmilk bank*”)<br>S1 (MH “Milk Banks”)
Web of Science Core Collection (SCI-Expanded; SSCI; ESCI; CPCI-S)	7 #5 AND #6<br>6 TS=("infant*" OR "infancy" OR "baby" OR "babies" OR "neonat*" OR "neo nat*" OR "newborn*" OR "new born*" OR "newly born*")<br>5 #3 OR #4<br>4 TS=("breastmilk donor*" OR "milk donat*" OR "breastmilk donat*" OR "breastmilk shar*" OR "milk shar*" OR "donor milk" OR "donor human milk" OR "donor breast milk" OR "donor breastmilk" OR "pasteurized human milk" OR "banked milk")<br>3 #2 AND #1<br>2 TS=("donor*" OR "donat*")<br>1 TS=("milk bank*" OR "breastmilk bank*")
Global Index Medicus (AIM; IMEMR; IMSEAR; LILACS; WPRO)	(mh:(infant) OR (ti:(infant* OR infancy OR baby OR babies OR neonat* OR "neo nat*" OR newborn* OR "new born*" OR "newly born*")) OR (ab:(infant* OR infancy OR baby OR babies OR neonat* OR "neo nat*" OR newborn* OR "new born*" OR "newly born*")) AND ((mh:("Milk Banks")) OR (ti:("milk bank" OR "milk banks" OR "milk banking" OR "breastmilk bank" OR "breastmilk banks" OR "breastmilk banking") OR (ab:("milk bank" OR "milk banks" OR "milk banking" OR "breastmilk bank" OR "breastmilk banks" OR "breastmilk banking"))) AND ((ti:(donor*)) OR (ab:(donor*)) OR ((ti:(donat*)) OR (ab:(donat*)))) OR (ti:("milk donation" OR "milk donations" OR "milk sharing" OR "donor milk" OR “donor human milk” OR “donor breast milk” OR “donor breastmilk" OR “pasteurized human milk”) OR (ab:("milk donation" OR "milk donations" OR "milk sharing" OR "donor milk" OR “donor human milk” OR “donor breast milk” OR “donor breastmilk" OR “pasteurized human milk”))))

Table S5. The impact of human milk donation on the donor: Table of excluded studies.

Study	Notes
Alencar 2009[2]	Reason(s) for exclusion:<br>- Wrong study design: This is a qualitative study.<br>- This study does not describe any results relating to our prespecified outcomes of interest.<br>- This study focuses mainly about the reasons why women donated and their sociodemographic profile. For the women interviewed who were former donors, rather than current donors, it does say the duration they donated and why they stopped (pain, lack of excess milk, return to work, etc.), but these are not our pre-specified outcomes of interest.

Asquith 1986[3]	Reason(s) for exclusion:<br>- Wrong study design: This is an opinion piece.<br>- This is a guideline about requirements for milk donation and best practice.
Balachandran 2018[4]	Reason(s) for exclusion:<br>- Wrong study design.<br>- No maternal outcomes of interest were reported.<br>- Postpartum hemorrhage could be of interest; however, it happens immediately in the first postnatal week. The mean donation started around nine days so the rates of postpartum hemorrhage would likely be unaffected by human milk donation.
Barbarska 2017[5]	Reason(s) for exclusion:<br>- Wrong study design: This is a descriptive study of milk donors.<br>- This study only discusses the sociodemographic profiles of the donors. There were no outcomes of interest investigated.
Candelaria 2018[6]	Reason(s) for exclusion:<br>- Wrong study design: This is a qualitative study.<br>- There were no controls, and no relevant outcomes reported.<br>- This study is about the motivation that drives donors to donate and their subjective experience while donating. None of our systematic review's pre-specified outcomes were reported.
Clifford 2022[7]	Reason(s) for exclusion:<br>- This study's reported outcomes are irrelevant to our prespecified outcomes of interest. This study reports events related to screening of donors in terms of their blood work.<br>- This is a poster. It only reports on adverse events related to blood draws.
Clifford 2022[8]	Reason(s) for exclusion:<br>- This study's outcomes were inconsistent with our prespecified outcomes of interest. The study mainly assessed the adverse effects related to phlebotomy for screening purposes and not the donation itself.<br>- No outcomes of interest were reported on.
Dambra-Candelaria 2017[9]	Reason(s) for exclusion:<br>- Wrong study design: This is a qualitative study.<br>- This study discusses why donors donate and how they feel towards donation.<br>- This study does not mention any of this systematic review's pre-specified outcomes.
Eidelman 2023[10]	Reason(s) for exclusion:<br>- Wrong study design: This is an opinion piece.
Fernández-Medina 2022[11]	Reason(s) for exclusion:<br>- This study's outcomes are not aligned with our prespecified outcomes. This study is about the effect of human milk donation on bereavement, which is not one of our prespecified outcomes.
Flores-Rojas 2023[12]	Reason(s) for exclusion:<br>- Wrong study design: This is a descriptive study about donors.<br>- The study is only a poster without full text available.

	- The study only reports on willingness to donate, rather than any donor-specific outcomes.
Freire 2022[13]	Reason(s) for exclusion:<br>- Wrong study design: This is a descriptive study on composition of milk.<br>- The weight change discussed in their pregnancy is from before pregnancy, which is a demographic variable, rather than an outcome.<br>- There is no mention of this systematic review's pre-specified outcomes.
Freire 2023[14]	Reason(s) for exclusion:<br>- Wrong study design: This study describes the association of maternal factors and certain metals in human milk.<br>- There were no outcomes reported for the effect of donation on the mother's health.<br>- This study reports the concentrations of metals in donated human milk in relation to donor's sociodemographic characteristics and dietary/environmental exposures.<br>- There is no mention of our pre-specified outcomes of interest.
GolubiÄ‡Ä†epuliÄ‡ 2020[15]	Reason(s) for exclusion:<br>- Wrong study design: The study appears to be a cross-sectional, descriptive analysis of donors.<br>- The abstract does not report the health outcomes for donor mothers.
Gutierrez Dos Santos 2023[16]	Reason(s) for exclusion:<br>- Wrong study design: The study is the description of donors and does not address the effect of donation on donors' health. This is about the profile of a donor.<br>- This study does not report any donor-specific outcomes.
Harris 2023[17]	Reason(s) for exclusion:<br>- This study's design is inconsistent with our review. The study does not describe the effect of donation on maternal outcomes. This is a descriptive study of who is donating and why.<br>- None of this systematic review's pre-specified outcomes were discussed.
Huerta 2020[18]	Reason(s) for exclusion:<br>- This study's design is inconsistent with our review. This study measures the motivation and not the effect of donation. The abstract seems to describe motivation. This is a descriptive study of donor profile and does not report any outcomes of interest.
Jaramillo-Ospina 2023[19]	Reason(s) for exclusion:<br>- The patient population is inconsistent with our review. Even though the study is non-English, the abstract clearly states that the authors studied the participants who were ineligible for donation.
Kadi 2020[20]	Reason(s) for exclusion:<br>- This study design is inconsistent with our review. This study is a descriptive study about donors. This study does not describe the effects of donation on the mother's health. This is a descriptive study regarding why mothers donate.<br>- None of this systematic review's pre-specified outcomes were mentioned.

Lagos Mendoza 2022[21]	Reason(s) for exclusion:<br>- Wrong study design: Even though the study seems to be in Spanish, the methods described in the methods described it as a qualitative study.
Machado 2015[22]	Reason(s) for exclusion:<br>- Wrong study design: This is a qualitative study.<br>- This study does not report on maternal mental health or other health. This is a descriptive study about why women donate and does not have any of our pre-specified outcomes.
Neves 2011[23]	Reason(s) for exclusion:<br>- Wrong study design: This is a qualitative study based on abstract.
Oliveira 2016[24]	Reason(s) for exclusion:<br>- Wrong study design: This study is a qualitative study about why mothers donate milk.
Oreg 2019[25]	Reason(s) for exclusion:<br>- The study's design is inconsistent with our review. The study is a review of previously published studies on bereavement. This is a descriptive study of why they donate.<br>- There were no pre-specified outcomes of interest mentioned.
Oreg 2023[26]	Reason(s) for exclusion:<br>- This study does not mention or include any of our pre-specified outcomes.<br>- This is an auto-ethnographical piece where authors share their traumatic experiences around childbirth and the healing that came from milk sharing. No pre-specified outcomes of interest were discussed.
Stokowski 2014[27]	Reason(s) for exclusion:<br>- The study's design is inconsistent with our review. This study discusses the risk-benefit ratio for the recipient infant, not the donor.
Tran 2023[28]	Reason(s) for exclusion:<br>- The study's design is inconsistent with our review. The study describes the factors associated with the production of human milk rather than the effect of donation on the mother's health.<br>- This study only discusses characteristics of those who donate milk; there were no pre-specified outcomes of interest discussed.
Tully 1999[29]	Reason(s) for exclusion:<br>- Wrong study design: This is a guideline.
Ward 2023[30]	Reason(s) for exclusion:<br>- Wrong study design: This is a descriptive analysis of experience of bereavement.<br>- This study did not discuss the effect on maternal outcomes.<br>- This study is about the emotional experience of donating human milk for bereaved donors; there were no pre-specified outcomes of interest discussed.
Welborn 2012[31]	Reason(s) for exclusion:<br>- Wrong outcomes: This is a descriptive, qualitative study of bereaved mothers.<br>- The study does not address the outcomes of this review.

	- This study is about the emotional experience of donating milk for bereaved donors and does not discuss any of our pre-specified outcomes of interest.
Widyaningrum 2022[32]	Reason(s) for exclusion:<br>- The study's design is inconsistent with our review. The study does not address the milk donors.<br>- This study does not describe the donor's outcomes, but rather women who do and do not receive donor human milk.

Table S6. The impact of human milk donation on the donor: Characteristics of included studies including population, exposure, comparison, and outcomes.

Study (Country)	Type of Study	Sample Size	Inclusion/Exclusion Criteria	Comparison Group	Outcome(s) Reported
**Azema 2003 (France)[33]**	Cross-sectional	103	Inclusion Criteria: Donating milk at one of eight participating milk banks; agreeing to participate and complete the survey.<br>Exclusion Criteria: Did not complete questionnaire.	No comparison group	Mastitis; engorgement; cracked/chapped nipples
**Osbaldiston 2007 (United States of America)[34]**	Retrospective Cohort	106	Inclusion Criteria for Donors: Participated/participating in donation at Mothers Milk Bank in Austin, TX, their donation occurred in prior 3 years, and consent was available.<br>Inclusion Criteria for Comparison Group: Mothers who breastfed or pumped milk for their infant within the prior 3 years and completed the consent form.<br>Exclusion Criteria: None provided.	Non-milk donor who had pumped milk while breastfeeding their infant over the prior 3 years	Postpartum depression and postpartum anxiety (reported as continuous outcome on 1-10 scale, did not extract); mastitis; engorgement; cracked/chapped nipples

**Mello-Neto 2009, 2010 (Brazil)**[35,36]	Cross-sectional	136	Inclusion Criteria: Donors at Marilia Human Milk Bank in Sao Paulo, Brazil between February 2003 and May 2004; healthy, breastfeeding or extracting human milk for their own child/children.<br>Exclusion Criteria: Smoking > 10 cigarettes/day; taking medicine incompatible with breastfeeding; using alcohol/illicit drugs.	No comparison group	Abnormal BMI; micronutrient deficiency (Vitamin A); exclusive breastfeeding
**Ahtiainen 2010 (Finland)**[37]	Retrospective Cohort	139	Inclusion Criteria: Women who had given birth at Tampere University Hospital between June 2007 and May 2008; milk donor or non-donor control mother randomly selected from a random sample of hospital birth records; provided voluntary informed consent.<br>Exclusion Criteria: Smoking; on regular medication; HIV/hepatitis infection; alcohol use.	Non-milk donors who gave birth to a live infant during the same time period	BMI; abnormal BMI; postpartum depression; postpartum anxiety; milk supply (but not mL/day, inadequate versus adequate, did not extract); mastitis; engorgement; cracked/chapped nipples
**Strambi 2012 (Italy)**[38]	Retrospective Cohort	200	Inclusion Criteria: Donor at one of the 5 Italian hospital milk banks included in the study.<br>Exclusion Criteria: None provided.	Non-milk donors	Abnormal BMI; pumped feeds; exclusive breastfeeding (reported as a categorical outcome, did not extract)
**Sierra-Colomina 2013, 2014 (Spain)**[39,40]	Prospective Descriptive	168	Inclusion Criteria: Accepted donor between December 2007 and June 2010 at the included milk bank; agreed to participate.<br>Exclusion Criteria: Did not complete questionnaire or return phone call.	No comparison group	Mastitis; engorgement; cracked/chapped nipples

**Olsson 2021 (Sweden)[41]**	Cross-sectional	72	Inclusion Criteria: Human milk donor during 2017 or 2018 at 2 referral university hospital milk banks; voluntarily agreeing to participate in the study.<br>Exclusion Criteria: None provided.	No comparison group	Mastitis; engorgement; cracked/chapped nipples (for all three, only stated that some reported this, did not specify quantity; authors were contacted, stated incidence data were not available)
**Peregoy 2022 (United States of America)[42]**	Cross-sectional	168	Inclusion Criteria: Aged 18 years or older; English-speaking; had engaged in milk sharing in the past 18 months (either as a donor or recipient); lived or worked in the DMV region (Washington, D.C.) at the time of milk sharing; provided informed consent.<br>Exclusion Criteria: None provided.	Human milk sharing recipient	Postpartum depression; postpartum anxiety; pumped milk feeding; breastfeeding duration (however, no data for each specific group; authors were contacted)
**Jayanandan 2024 (India)[43]**	Cross-sectional	140	Inclusion Criteria: Postnatal mother of child < 6 months of age; registered as either a donor or a recipient of the milk bank.<br>Exclusion Criteria: None provided.	Human milk bank recipient	Milk supply (but not mL/day, self-reported insufficiency, did not extract)

Table S7. The impact of human milk donation on the donor: Maternal and infant characteristics of the included studies.

Study (Country)	Recruitment of Human Milk Bank (HMB) Donors	Maternal Age of HMB Donors (Age of non-donors)	Motivations to Donate Human Milk	Gestational Age of Infant	Birth Weight of Infant	Duration/Volume of Human Milk Donation	Brief Findings

**Azema 2003 (France)[33]**	Current Donors at Eight HMBs	Average Age of Donors:<br>30.6 years old (SD = 4.45 years); Range: 20-42 years old	71.8% of donors reported this was their first time donating<br><br>Reasons for Donating:<br>Nearly 60% of donors indicated "having too much milk" as a primary, secondary, or tertiary reason for donating<br><br>Second most common reason for donating as a desire to help others	Did Not Report	Did Not Report	Did Not Report	19.4% of donors reported engorgement during breastfeeding<br><br>10.7% of donors reported cracked or chapped nipples during breastfeeding<br><br>1.9% of donors reported mastitis during breastfeeding<br><br>19.4% of donors reported experiencing more than 1 of the listed problems above
**Osbaldiston 2007 (United States of America)[34]**	Established Donors at HMB	25-29 years old: 18% of donors (non-donors: 26%)<br><br>30-34 years old: 48% of donors (non-donors: 37%)<br><br>35-39 years old: 25% of donors (non-donors: 32%)	How donors heard about breastmilk donation:<br>Healthcare professionals or healthcare setting (17%)<br>Friends (28%)<br>Website, TV, radio (22%)<br>Other (31%)<br><br>Four highest reasons for donating:<br>To help others<br>Had too much milk and wanted to donate it<br>Know the milk bank needs donations<br>Would hope someone would do the same if I were in need	Did Not Report	Did Not Report	Average amount of human breastmilk donated after excluding two outliers: 29.65 +/- 31.13L	58% of donors reported breast engorgement<br><br>33% of donors reported cracked or chapped nipples<br><br>20% of donors reported a breast infection or mastitis<br><br>Incidence of depression identified as a barrier to donating breastmilk in donors was 0.26 (+/- 1.26) and in non-donors was 1.87 (+/- 3.04)
**Mello-Neto 2009, 2010 (Brazil)[35,36]**	Current Donors at HMB	16-19 years old: 13.2%<br><br>20-29 years old: 54.5%<br><br>30-41 years old: 32.4%	Did Not Report	Did Not Report	Did Not Report	Did Not Report	3.1% of donors were classified as malnourished (BMI 17-18.4), 54% of donors had a normal BMI (BMI 20-24.99), 31% of donors were classified as pre-obese (BMI 25 -29.9), and 11.8% were classified as obese (BMI 30 - 41)<br><br>81.6% of donors were exclusively breastfeeding<br><br>25.7% of donors had concentrations of vitamin A in

							their blood that was < 0.07 umol/L
**Ahtiainen 2010 (Finland)**[37]	Past Donors at HMB	Average Age of HMB Donors: 32.3 years old (SD = 5.1)<br><br>Average Age of Non-donors: 32.8 years old (SD = 4.7)	Where Donors Received Information about HMBs:<br>Healthcare setting (73%)<br>From a friend (16%)<br>Internet/TV/Radio (10%)<br><br>Top Reasons for Donating:<br>Desire to help others<br>Unwillingness to throw milk away<br>Need for donated milk	Median Gestational Age of Donor Infants: 40+0 weeks (IQR: 38+1 – 40+4)<br><br>Median Gestational Age of Non-donor Infants: 40+3 weeks (IQR: 39+1 – 41+0)	Median Birth Weight of Donor infants: 3,537 g (IQR: 3,034-3,852 g)<br><br>Median Birth Weight of Non-donor Infants: 3,450 g (IQR: 3,160-3,975 g)	Median Cumulative Amount of Human Breastmilk Donated: 12.6L	20% of donors perceived inadequate milk supply compared to 41% of non-donors ($p = 0.008$)<br><br>22% of donors reported a breast infection compared to 15% of non-donors ($p = 0.286$)<br><br>19% of donors reported cracked or chapped nipples compared to 30% of non-donors ($p = 0.107$)<br><br>29% of donors reported breast engorgement compared to 15% of non-donors ($p = 0.062$)<br><br>25% of donors reported feeling somewhat anxious compared to 37% of non-donors ($p > 0.05$) *<br><br>32% of donors reported experiencing depression compared to 32% of non-donors ($p = 0.971$)<br><br>Median BMI for donors was 23.8 (IQR: 22-28) compared to 23.7 (IQR: 21-26) for non-donors ($p = 0.262$)<br><br>41% of donors demonstrated a BMI > 25, further classified as abnormal compared to 32%

							of non-donors (*p* = 0.290)
**Strambi 2012 (Italy)[38]**	Current Donors at Five HMBs	22-26 years old: 6% (non-donors: 4%)<br><br>27-29 years old: 28% (non-donors: 28%)<br><br>30-34 years old: 18% (non-donors: 19%)<br><br>35-36 years old: 39% (non-donors: 38%)<br><br>37-44 years old: 9% (non-donors: 11%)	73% of donors found out about breast milk donation from healthcare workers<br><br>19% of donors had previously donated human breast milk<br><br>9% of donors previously utilized donated breast milk for a child of theirs	Preterm Infants: 12% (non-donors: 27%)<br><br>Non-preterm Infants >37 and <41 Weeks Gestation: 81% (non-donors: 68%)<br><br>Non-preterm Infants >41 Weeks Gestation: 7% (non-donors: 5%)	Birth Weight <2 kg: 10% (non-donors: 13%)<br><br>Birth Weight >2 and <2.9 kg: 29% (non-donors: 28%)<br><br>Birth Weight > 3 kg and <3.9 kg: 56% (non-donors: 54%)<br><br>Birth Weight >4 kg: 5% (non-donors: 5%)	Did Not Report	42% of donors reported abnormal BMI (14% underweight and 28% overweight or obese) compared to 64% of non-donors (6% underweight and 58% overweight or obese)<br><br>72% of donors reported having to pump breastmilk to feed their child compared to 58% of non-donors
**Sierra-Colomina 2013, 2014 (Spain)[39,40]**	Current and Recent Donors at HMB	Average Age of Donors:<br>33.1 +/- 4.5 years old; Range: 18-42 years old	Reasons for Donating:<br>Excess milk and preferred to donate as opposed to throw away (77%)<br>Help others (75%)<br>I hope someone would do the same for me if I needed it (16%)<br>I know someone who personally has a newborn who needs donated milk (10%)	Average Gestational Age of Donor Infants: 37.8 weeks +/- 3.6 weeks; Range: 24-42 weeks	Average Birth Weight of Donor Infants: 2,975 g +/- 835 g; Range: 780-4,800 g	Average Time of Human Breastmilk Donation: 189 days +/- 135 days	Nine of the 98 donors reported mastitis<br><br>Six of the 98 donors reported cracked or chapped nipples<br><br>Four of the 98 donors reported breast engorgement
**Olsson 2021 (Sweden)[41]**	Current or past donors at two HMBs	Average Age of Donors: 32 years old; Range: 24-41 years old	15.3% of donors had previously donated breast milk<br><br>Reasons for Donating:<br>Strong desire to help other infants<br>Prior experiences with older children or children of family/friends who needed donor breast milk	Preterm Infants: 31% (further defined as born after an average gestational age of 30.4 weeks [25-35]; SD 2.95)<br><br>Full-term Infants: 69%	Did Not Report	Average Duration of Donation Period:<br>8.7 weeks (SD 5.94); Range: 1-24 weeks	Participant reports of sore nipples, mastitis, and pain in the breasts due to an increasing excess of milk from expression.<br><br>No numerical data available.

**Peregoy 2022 (United States of America)[42]**	Current or past participants (either donor or recipient) of milk-sharing	18-34 years old: 67.4% (recipients: 48.6%)<br><br>35-44 years old: 32.6% (recipients: 50%)<br><br>45-54 years old: 0% (recipients: 1.5%)	Means of Connecting with Human Milk-sharing Parents: Online group Already knew the parents Facilitated through mutual friend, lactation consultant, or midwife/doula<br><br>5.1% of donors had received breast milk from a HMB, 4.1% of donors had ever had their baby cross-nursed by another person, and 3.1% of donors had ever received shared human milk or purchased human milk	28-31 Weeks Gestation: 3.1% (recipients: 2.9%)<br><br>32-36 Weeks Gestation: 7.1% (recipients: 7.1%)<br><br>37+ Weeks Gestation: 89.8% (recipients: 90.0%)	Did Not Report	55% of respondents exchanged a total of less than 250 ounce of shared human breast milk, while 22% exchanged 1000 or more ounces	13.4% of donors experienced postpartum depression compared to 38.2% of recipients**<br><br>40.2% of donors experienced postpartum anxiety compared to 41.2% of recipients<br><br>96.9% of donors pumped breast milk at some point to feed their child compared to 66% of recipients<br><br>Median duration of breast milk feeding did not significantly differ between donors and recipients
**Jayanandan 2024 (India)[43]**	Current or recent donor mothers and current or recent recipient mothers at HMB	Average Age of Donors: 25.6 years old (SD = 3.9 years); Range: 18-36 years old<br><br>Average Age of Recipients: 25.9 years old (SD = 4.5 years); Range: 15-35 years old	100% of donors noted awareness of HMBs<br><br>94.3% of donors identified their source of information on HMBs were staff nurses<br><br>98.6% of donors shared willingness to donate breast milk in the future	Did Not Report	Very Low Birth Weight (< 1.500 kg): 4.3% of donor infants 11.4% of recipient infants<br><br>Low Birth Weight (1.500-2.499 kg): 34.3% of donor infants 30.0% of recipient infants<br><br>Normal Birth Weight (>/= 2.500 kg): 61.4% of donor infants 58.6% of recipient infants	Median Quantity of Milk Donated: 50 mL (IQR: 25-75 mL; Range: 5-150 mL)	None of the included donors reported any side effects or milk insufficiency after donation

*Did not report exact p-value, rather reported "no significant difference"
**p<0.0001

Figure S1: Risk of Abnormal BMI - Overweight. This forest plot demonstrates the risk of having an overweight BMI associated with human milk donation.

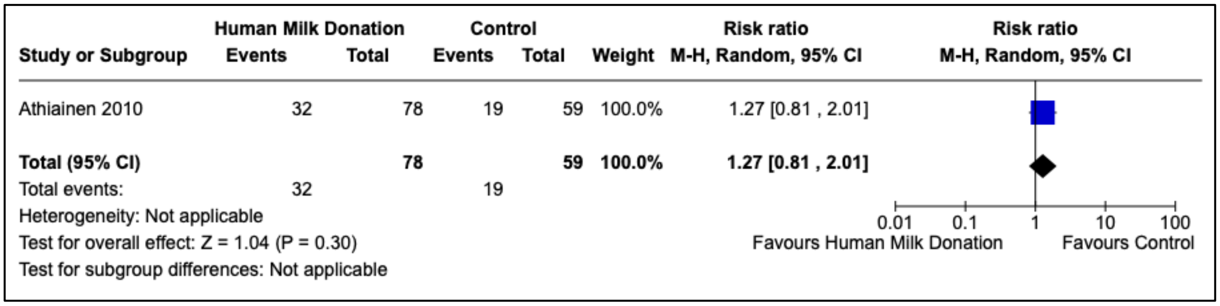

Table S8. The impact of human milk donation on the donor: Outcomes of interest from studies lacking information necessary for further analysis.

**Outcome**		**Findings**
**Health, Nutritional, and Psychosocial Outcomes**	Abnormal BMI - Underweight	One study reported on the incidence of underweight BMI, a primary nutritional outcome, in donors with 2% of donors being underweight.[35,36] The study did not include a comparison group.
	Vitamin A Deficiency	One study reported on the incidence of vitamin A deficiency, a primary nutritional outcome, in the human milk donor group (25%), but the study did not include a comparison group.[35,36]
**Well-being, Prevention of Harm, Lactation Experience, and Additional Outcomes**	Breastfeeding Exclusivity	One study reported on this outcome; 82% of donors reported they exclusively breastfed their infants (No. of donors: 136).[35,36]
	Breastfeeding Duration	One study reported on this outcome; the median breastfeeding duration among the donor group was 13.5 months which was longer than the median breastfeeding duration of 12.0 months in the non-donor group.[42]<br><br>We could not calculate a summary estimate because of the lack of interquartile range

		for the comparison group. The authors were contacted for this information, and we did not receive a response.

Figure S2: Risk of Pumped Milk Feeding. This forest plot demonstrates the risk of needing to feed one's own infant pumped milk associated with human milk donation.

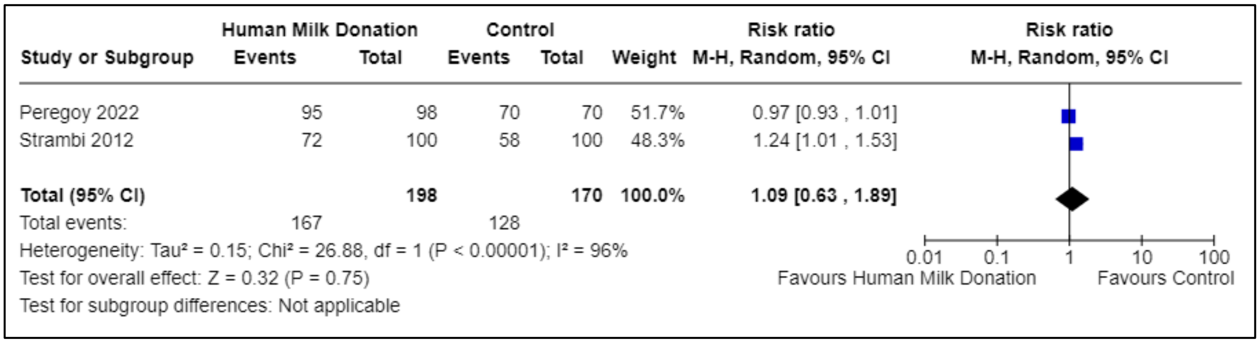

Table S9. The impact of human milk donation on the donor's infant: Table of excluded studies.

Study	Notes
Peregoy 2022[42]	Reason(s) for Exclusion:<br>- The study's design was inconsistent with our review's parameters. This study did not report on infant outcomes.
Luginina-Kovalevskaia 1968[44]	Reason(s) for Exclusion:<br>- This study included a patient population that was irrelevant to our review. This study investigated outcomes in infants who received donated breast milk rather than infants of mothers who donated human breast milk.

Table S10. The impact of human milk donation on the donor infant: Characteristics of included studies including population, exposure, comparison, and outcomes.

Study (Country)	Type of Study	Setting	Population Size	Population Selection	Exposure Group	Comparison Group	Method of Data Collection	Outcomes of Interest

**Ahtiainen 2010 (Finland)[37]**	Retrospective Cohort	HMB at Public University Hospital in Finland	N =139 infants<br><br>Infants of donors = 58%	Random selection from women who birthed a live infant at the hospital during June 2007 - May 2008 and had either donated their breast milk (exposure group) or had not donated their breast milk (comparison group). No detailed exclusion criteria.	Human Milk Donation	Non-human milk donors and their infants	Mailed questionnaire and health records	Thrush; reflux; slow weight gain; need for phototherapy; weight loss.
**Balachandran 2018 (India)[4]**	Cross-sectional	HMB within Tertiary NICU in India	N = 217 infants<br><br>Infants of donors = 100%	Women with an infant in the NICU who had donated for >30% of their hospital stay were approached for inclusion in this study.	Human Milk Donation	No comparison	Utilized milk bank database and hospital records	Infant mortality
**Osbaldiston 2007 (United States of America)[34]**	Retrospective Cohort	Non-profit HMB in the United States of America	N = 106 infants<br><br>Infants of donors = 82%	Women in Texas who had donated their breast milk to this bank in the past 3 years.	Human Milk Donation	Non-human milk donors who had pumped milk while breastfeeding their infant over the last 3 years and their infant	Telephone survey	Thrush; reflux; slow weight gain
**Sierra-Colomina 2014 (Spain)[40]**	Cross-sectional	University Hospital HMB in Spain	N = 415 infants<br><br>Infants of donors = 100%	All women who donated their breast milk between January 2009 - April 2013 and had finished the donation process were included. Women who did not meet eligibility criteria for donation were excluded.	Human Milk Donation	No comparison	HMB database	Infant mortality
**Olsson 2021 (Sweden)[41]**	Cross-sectional	Two Referral University Hospital HMBs in Sweden	N = 72 infants<br><br>Infants of donors = 100%	Women who donated their breast milk during 2017-2018 and voluntarily agreed to participate. No explicit exclusion criteria listed.	Human Milk Donation	No comparison	Mailed survey	Feeding Intolerance (diarrhea)

**Sierra-Colomina 2013 (Spain)[39]**	Cross-sectional	Single HMB in Spain	N = 168 infants initially, with 98 infants being included in all steps of study<br><br>Infants of donors = 100%	Women who donated their breast milk between December 2007 - June 2010 at the HMB of interest who consented to participation. Women were excluded if they did not complete initial questionnaire or if they did not answer the telephone for the second component.	Human Milk Donation	No comparison	Written questionnaire, follow-up telephone interview	Slow weight gain

Table S11. The impact of human milk donation on the donor infant: Maternal and infant characteristics of the included studies' populations.

Study (Country)	Recruitment of HMB Donors	Maternal Age of HMB Donors	Gestational Age of Infant at Birth	Birth Weight of Infant	Age of Infant at Initiation of HMB Donation	Duration/Volume of HMB Donation	Brief Findings
**Ahtiainen 2010 (Finland)[37]**	Past donors at HMB	Average Age of Donors: 32.3 years (SD = 5.1)	Median gestational age of included infants: 40 + 0 weeks (IQR: 38+1 - 40+4)	Median birth weight of included infants: 3537 g (IQR: 3034-3852 g)	Donation was initiated within two months of infant delivery and could have been continued until three months post-delivery, per this HMB's protocol	Median cumulative amount of human milk donated: 12.6 L	The need of phototherapy was significantly more common and post-natal weight loss was greater in infants of donors compared to infants of non-donors. Slow weight gain was significantly more common in infants of non-donors compared to infants of donors. No significant difference in the presence of thrush or reflux among infants of donors and non-donors.

**Balachandran 2018 (India)[4]**	Established donors at HMB	< 18 years old: 2.76% of donors<br><br>18-24 years old: 14.26% of donors<br><br>25-35 years old: 81.11% of donors<br><br>> 35 years old: 1.84% of donors	< 34 weeks: 19.35% of infants<br><br>34-37 weeks: 20.74% of infants<br><br>38-41 weeks: 52.99% of infants<br><br>> 41 weeks: 6.91% of infants	< 1000 g: 12.44% of infants<br><br>1000-1499 g: 27.19% of infants<br><br>1500-2499 g: 42.86% of infants<br><br>>/= 2500 g: 18.89% of infants	Average age of infant: 9 days old (SD = 3.47 days)	No explicit amount indicated	Infant mortality among infants of human milk donors was reported to be 14.75%.
**Osbaldiston 2007 (United States of America)[34]**	Established donors at HMB	25-29 years old: 18% of donors<br><br>30-34 years old: 48% of donors<br><br>35-39 years old: 25% of donors	Did not report	Did not report	Did not report	Average amount of human milk donated after excluding two outliers: 29.65 L +/- 31.13 L	13% of infants of human milk donors were reported to have thrush, 6% were reported to have slow weight gain, and 25% were reported to have reflux.
**Sierra-Colomina 2014 (Spain)[40]**	Past donors at HMB	Median age of donors: 33.6 years (IQR: 31-36 years)	< 32 weeks: 9.9% of infants<br><br>32-36 weeks: 13.2%<br><br>> 37 weeks: 76.9%	Did not report	Median age of infants: 2.9 months (IQR: 1.3-5. 7 months)<br><br>Range: 0-28 months	Median volume of human milk donated per woman: 3.1 L	Infant mortality among infants of human milk donors was reported to be 4.1%.
**Olsson 2021 (Sweden)[41]**	Current or past donors at two HMBs	Average age of donors: 32 years old<br><br>Range: 24 - 41 years old	Preterm Infants: 31% and further defined as born after an average gestational age of 30.4. weeks ([25-35]; SD = 2.95)<br><br>Full term infants: 69%	Did not report	Average age of infant: 3.8 weeks (SD = 3.23)	Average duration of donation period: 8.7 weeks (SD = 5.94)<br><br>Range: 1-24 weeks	One human milk donor shared that her infant frequently had diarrhea due to her increased milk production.
**Sierra-Colomina 2013 (Spain)[39]**	Current donors at HMB	Average age of donors: 33.1 +/- 4.5 years old<br><br>Range: 18-42 years old	Average: 37.8 weeks +/- 3.6 weeks; Range: 24-42 weeks	Average birth weight: 2,975 +/- 835 g<br><br>Range: 780-4,800 g	Average infant age: 129 +/- 142 days	Average Duration of Human Milk Donation: 189 days +/- 135 days	1% of infants of human milk donors experienced slow weight gain.

Table S12. The impact of human milk donation on the donor infant: Further outcomes of interest.

Outcome		Findings
**Health Outcomes**	Feeding Intolerance (diarrhea)	One study without data available from the comparison group reported on the primary health outcome of feeding intolerance (diarrhea).[41] This study included one participant who spontaneously reported their infant experienced frequent diarrhea due to the increased human milk production given they were simultaneously breastfeeding and expression.
**Growth, Development, and Additional Outcomes**	Percent Weight Loss After Birth	One study reported on the additional outcome of interest of percent weight loss after birth.[37] This study included a total of 139 infants, with 80 in the donor infant group and 59 in the comparison infant group. There were data for this outcome from 64 donor infants and 57 non-donor infants with slightly more percent weight loss in the group with human milk donation compared to the comparison group (mean difference: 0.72, 95% CI [-1.42, -0.01].

References

1. Berg A, Rani U, Colaizy T, et al. The impact of donating milk on the health of milk donors and their infants: A systematic review and meta-analysis protocol. *JPGN Reports*. 06/24 2024:n/a-n/a. doi:10.1002/jpr3.12101
2. Alencar LC, Seidl EM. Breast milk donation: women's donor experience. *Rev Saude Publica*. 2009;43(1):70-7. doi:10.1590/s0034-89102009000100009
3. Asquith MT. Human Milk Banks: The Donor. *Journal of Human Lactation*. 1986;2(1):20-22. doi:10.1177/089033448600200106
4. Balachandran AM, C. N. K, Bharathi S. M. Sociodemographic and clinical profile of human milk donors and their infants in a model human milk bank: a descriptive cross-sectional study. *International Journal of Contemporary Pediatrics*. 2018;5(5):6. doi:http://dx.doi.org/10.18203/2349-3291.ijcp20183368
5. Barbarska O, Zielińska M, Pawlus B, Wesołowska A. Characteristics of the regional human milk bank in Poland - donors, recipients and nutritional value of human milk. *Rocz Panstw Zakl Hig*. 2017;68(4):395-400.

6. Candelaria LM, Spatz DL, Giordano N. Experiences of Women Who Donated Human Milk. *J Obstet Gynecol Neonatal Nurs*. 2018;47(4):556-563. doi:10.1016/j.jogn.2017.12.007
7. Clifford V, Klein L, Brown R, et al. DONOR AND RECIPIENT ADVERSE EVENTS IN MILK BANKING. *Journal of Paediatrics and Child Health*. 2022;58(SUPPL 2):54. doi:10.1111/jpc.15945
8. Clifford V, Klein LD, Brown R, et al. Donor and recipient safety in human milk banking. *J Paediatr Child Health*. 2022;58(9):1629-1634. doi:10.1111/jpc.16066
9. Dambra-Candelaria LM. Mothers Voices: The Lived Experience of the Human Milk Banking Association of North America Milk Donor. *Mothers Voices: Lived Experience of the Human Milk Banking Association of North America Milk Donor*. 2017:1-1.
10. Eidelman AI. Breastfeeding and the Bereaved Parent. *Breastfeeding Medicine*. 2023;18(4):253-253. doi:10.1089/bfm.2023.29242.editorial
11. Fernández-Medina IM, Jiménez-Lasserrotte MDM, Ruíz-Fernández MD, Granero-Molina J, Fernández-Sola C, Hernández-Padilla JM. Milk Donation Following a Perinatal Loss: A Phenomenological Study. *J Midwifery Womens Health*. 2022;67(4):463-469. doi:10.1111/jmwh.13342
12. Flores-Rojas K, Pastor-Villaescusa B, Lacort Peralta I, Gil-Campos M. Functional Feasibility Study of a Donated Milk Bank in a Third Level Hospital. *Annals of Nutrition and Metabolism*. 2023;79(1):79. doi:10.1159/000526958
13. Freire C, Iribarne-Durán LM, Gil F, et al. Concentrations and determinants of lead, mercury, cadmium, and arsenic in pooled donor breast milk in Spain. *Int J Hyg Environ Health*. 2022;240:113914. doi:10.1016/j.ijheh.2021.113914
14. Freire C, Iribarne-Durán LM, Gil F, et al. Concentrations and predictors of aluminum, antimony, and lithium in breast milk: A repeated-measures study of donors. *Environ Pollut*. 2023;319:120901. doi:10.1016/j.envpol.2022.120901
15. Golubić Ćepulić B, Novoselac J, Pavičić Bošnjak A, Leskovar I, Gojčeta K, Rimac V. Human Milk Bank in Croatia: initial experiences. *Paediatria Croatica*. 2020;64(2):103-110. doi:10.13112/PC.2020.15
16. Gutierrez Dos Santos B, Shenker N, Weaver G, Perrin MT. Comparison of Breastfeeding and Pumping Experiences of Milk Bank Donors in the United States and United Kingdom. *Breastfeed Med*. 2023;18(11):870-880. doi:10.1089/bfm.2023.0172
17. Harris S, Bloomfield FH, Muelbert M. HUMAN MILK DONATION in AOTEAROA NEW ZEALAND: SURVEY of MOTHERS and HEALTH PROFESSIONALS. *Journal of Paediatrics and Child Health*. 2023;59:82. doi:10.1111/jpc.16357
18. Huerta CT, Vergara DM, Dávila FP, Leal FS, Hidalgo CC. Characteristics and motivations of donors to the human milk ba nk in Dr. Sótero Del Rio hospital, Chile. *Revista Chilena de Nutricion*. 2020;47(1):105-113. doi:10.4067/S0717-75182020000100105
19. Jaramillo-Ospina AM, Acevedo-Castaño I, Castillejo-Padilla NP, Mazo-Calle NR. Modifiable factors associated with loss of donors in a human milk bank. *Revista de Salud Publica*. 2023;25(1)doi:10.15446/rsap.v25n1.104729
20. Kadi H, Lamireau D, Bouncer H, et al. Satisfaction of mothers regarding human milk donation. *Arch Pediatr*. 2020;27(4):202-205. doi:10.1016/j.arcped.2020.03.005
21. Lagos Mendoza SL, Lombo Caicedo JC, Soto Morales AM, Rubio LS. Donating human milk saves lives: insights from women donors and recipients of milk at a human

milk bank in Colombia. *Cultura de los Cuidados*. 2022;26(64):122-135. doi:10.14198/cuid.2022.64.11
22. Machado RDS, Campos Calderón CP, Montoya Juárez R, Schmidt RioValle J. Experiences of human milk donation in Andalucía-Spain: a qualitative study. *Enfermeria Global*. 2015;14(1):125-135. doi:10.6018/eglobal.14.1.192401
23. Neves LS, Sá MVM, Mattar MJG, Galisa MS. Human milk donation: Difficulties and limiting factors. *Mundo da Saude*. 2011;35(2):156-161.
24. Oliveira MMBd. A representação social de mulheres doadoras de leite humano. 2016. p. 135 p-135 p.
25. Oreg A. Milk donation after losing one's baby: Adopting a donor identity as a means of coping with loss. *Soc Sci Med*. 2019;238:112519. doi:10.1016/j.socscimed.2019.112519
26. Oreg A, Perez AS, Timor-Shlevin S. "So, whose milk was it? ... It became all of ours, together": A relational autoethnographic study of an interactional human milk donation process through bereavement. *Death Stud*. 2023;47(8):938-947. doi:10.1080/07481187.2022.2143936
27. Stokowski LA. DONOR HUMAN MILK: THE RISK-BENEFIT RATIO. *Advances in Neonatal Care*. 2014;14(4):237-237.
28. Tran HT, Nguyen TT, Nguyen OTX, Barnett D, Weaver G, Mathisen R. Characteristics and factors influencing the volume of breastmilk donated by women to the first human milk bank in Vietnam. *Front Glob Womens Health*. 2023;4:1185097. doi:10.3389/fgwh.2023.1185097
29. Tully MR. Donating human milk as part of the grieving process. *J Hum Lact*. 1999;15(2):149-51. doi:10.1177/089033449901500217
30. Ward G, Adair P, Doherty N, McCormack D. Bereaved mothers' experience of expressing and donating breast milk: An interpretative phenomenological study. *Matern Child Nutr*. 2023;19(3):e13473. doi:10.1111/mcn.13473
31. Welborn JM. The experience of expressing and donating breast milk following a perinatal loss. *J Hum Lact*. 2012;28(4):506-10. doi:10.1177/0890334412455459
32. Widyaningrum R, Wienarno E, Khofifah H, Herliyanti Y, Wahyuni Z. THE CORRELATION AMONG HUMAN MILK DONOR, BOTTLE FEEDING, AND BREASTFEEDING STATUS OF MOTHER OF 0-6 MONTHS INFANTS IN YOGYAKARTA, INDONESIA. *Journal of Public Health Research & Community Health Development*. 2022;6(1):75-81. doi:10.20473/jphrecode.v6i1.30209
33. Azema E, Callahan S. Breast milk donors in France: a portrait of the typical donor and the utility of milk banking in the French breastfeeding context. *J Hum Lact*. May 2003;19(2):199-202. doi:10.1177/0890334403252476
34. Osbaldiston R, Mingle LA. Characterization of human milk donors. *J Hum Lact*. Nov 2007;23(4):350-7; quiz 358-61. doi:10.1177/0890334407307547
35. Mello-Neto J, Rondo PH, Oshiiwa M, Morgano MA, Zacari CZ, Domingues S. The influence of maternal factors on the concentration of vitamin A in mature breast milk. *Clin Nutr*. Apr 2009;28(2):178-81. doi:10.1016/j.clnu.2009.02.002
36. Mello-Neto J, Rondo PH, Morgano MA, Oshiiwa M, Santos ML, Oliveira JM. Iron concentrations in breast milk and selected maternal factors of human milk bank donors. *J Hum Lact*. May 2010;26(2):175-9. doi:10.1177/0890334409353748

37. Ahtiainen K, Luukkaala T, Tammela O. Mother- and Infant-Dependent Factors Influencing Breast Milk Donation. *Pediatric Research*. 2010/11/01 2010;68(1):506-506. doi:10.1203/00006450-201011001-01017
38. Strambi M, Anselmi A, Coppi S. [Donors' personal profile in Tuscany's network of milk banks]. *Minerva Pediatr*. Oct 2012;64(5):501-11. Il profilo personale delle donatrici nella rete toscana delle banche del latte umano donato.
39. Sierra Colomina G, García Lara N, Escuder Vieco D, Vázquez Román S, Cabañes Alonso E, Pallás Alonso CR. Características de las mujeres donantes de un banco de leche materna y relación con el tiempo de donación. *Anales de Pediatría*. 2014/04/01/ 2013;80(4):236-241. doi:https://doi.org/10.1016/j.anpedi.2013.05.017
40. Sierra-Colomina G, García-Lara NR, Escuder-Vieco D, Alonso-Díaz C, Esteban EM, Pallás-Alonso CR. Donor milk volume and characteristics of donors and their children. *Early Hum Dev*. May 2014;90(5):209-12. doi:10.1016/j.earlhumdev.2014.01.016
41. Olsson E, Diderholm B, Blomqvist YT. "Paying it Forward" - Swedish Women's Experiences of Donating Human Milk. *J Hum Lact*. Feb 2021;37(1):87-94. doi:10.1177/0890334420979245
42. Peregoy JA, Pinheiro GM, Geraghty SR, Dickin KL, Rasmussen KM. Human milk-sharing practices and infant-feeding behaviours: A comparison of donors and recipients. *Matern Child Nutr*. Oct 2022;18(4):e13389. doi:10.1111/mcn.13389
43. Jayanandan R, Rajamanickam R, Kumaran R, et al. Perception and Practices Related to Breast Milk Donation and Acceptance among Donor and Recipient's Mothers of a Breast Milk Bank at a Tertiary Care Hospital in South India. *Indian Journal of Public Health and Research Development*. 2024;15(1):288-293. doi:https://doi.org/10.37506/ewvys375
44. Luginina-Kovalevskaia NM. [Physical development and morbidity in children, whose mothers served as donors and in those fed with donor milk]. *Vopr Okhr Materin Det*. Jan 1968;13(1):89-90. Fizicheskoe rezvitie i zabolevaemost' deteĭ, materi kotorykh byli donorami, i deteĭ, pitavshikhsia donorskim molokom.
